# Tumor-initiating cells of breast and prostate origin show alterations in the expression of genes related to iron metabolism

**DOI:** 10.18632/oncotarget.14093

**Published:** 2016-12-22

**Authors:** Zuzana Rychtarcikova, Sandra Lettlova, Veronika Tomkova, Vlasta Korenkova, Lucie Langerova, Ekaterina Simonova, Polina Zjablovskaja, Meritxell Alberich-Jorda, Jiri Neuzil, Jaroslav Truksa

**Affiliations:** ^1^ Institute of Biotechnology, Czech Academy of Sciences, Prague, Czech Republic; ^2^ Charles University in Prague, Faculty of Pharmacy in Hradec Kralove, Hradec Kralove, Czech Republic; ^3^ Charles University in Prague, Faculty of Sciences, Prague, Czech Republic; ^4^ Institute of Molecular Genetics, Czech Academy of Sciences, Prague, Czech Republic; ^5^ School of Medical Science, Menzies Health Institute Queensland, Southport, Queensland, Australia

**Keywords:** tumor-initiating cells, breast cancer, iron metabolism, FeS cluster, stem cells

## Abstract

The importance of iron in the growth and progression of tumors has been widely documented. In this report, we show that tumor-initiating cells (TICs), represented by spheres derived from the MCF7 cell line, exhibit higher intracellular labile iron pool, mitochondrial iron accumulation and are more susceptible to iron chelation. TICs also show activation of the IRP/IRE system, leading to higher iron uptake and decrease in iron storage, suggesting that level of properly assembled cytosolic iron-sulfur clusters (FeS) is reduced. This finding is confirmed by lower enzymatic activity of aconitase and FeS cluster biogenesis enzymes, as well as lower levels of reduced glutathione, implying reduced FeS clusters synthesis/utilization in TICs. Importantly, we have identified specific gene signature related to iron metabolism consisting of genes regulating iron uptake, mitochondrial FeS cluster biogenesis and hypoxic response (*ABCB10*, *ACO1*, *CYBRD1*, *EPAS1*, *GLRX5*, *HEPH*, *HFE*, *IREB2*, *QSOX1* and *TFRC*). Principal component analysis based on this signature is able to distinguish TICs from cancer cells *in vitro* and also Leukemia-initiating cells (LICs) from non-LICs in the mouse model of acute promyelocytic leukemia (APL). Majority of the described changes were also recapitulated in an alternative model represented by MCF7 cells resistant to tamoxifen (TAMR) that exhibit features of TICs. Our findings point to the critical importance of redox balance and iron metabolism-related genes and proteins in the context of cancer and TICs that could be potentially used for cancer diagnostics or therapy.

## INTRODUCTION

Iron is an indispensable micro-nutrient, on which most living organisms depend for their existence due to its ability to shuffle between ferrous and ferric form and participate in crucial redox reactions. Iron also serves as a metal cofactor needed for DNA synthesis and repair as well as a cofactor of many metabolic enzymes and enzymes of the respiratory complexes in mitochondria [1, 2, 3].

There are many iron metabolism-related proteins that play pivotal role in cellular iron handling, for example proteins related to iron uptake such as transferrin receptor (TFR1 encoded by the *TFRC*) and cytochrome b reductase (coded by *CYBRD1*) [3, 4] as well as regulators participating in iron storage such as ferritin encoded by the *FTH* and *FTL* genes [5]. Additional proteins participating in the iron utilization and FeS cluster assembly are glutaredoxin 5 (encoded by *GLRX5*) and ATP binding cassette subfamily B member 10 (coded by *ABCB10*) [1, 6, 7]. There are other critical enzymes participating in the iron sensing such as iron responsive protein 1 (IRP1) encoded by the aconitase (*ACO1)* gene and iron responsive protein 2 (IRP2) coded by the iron responsive element binding protein 2 (*IREB2*) gene [8]. These proteins play a crucial role in assessing the intracellular iron level and eliciting appropriate response by modulating iron uptake and iron storage *via* binding to the iron responsive elements (IRE) located at the 5´and 3´prime untranslated regions of the corresponding mRNA [9]. Furthermore, there is a tight crosstalk between the hypoxic response of the cell and cellular iron metabolism as low iron levels elicit activation of the hypoxia inducible factors (HIF) encoded by the *HIF1A* and endothelial Per-ARNT-Sim Domain Protein 1 (*EPAS1*) genes [10]. Activation of these genes is connected with higher iron uptake through CYBRD1 in the enterocytes [11] and in the non-physiological setting it is connected with activation of tissue remodelling factors such as quiescin sulfhydryl oxidase1 (coded by *QSOX1)* [12]. Other important regulators of iron metabolism represent proteins involved in iron export such as hephaestin encoded by the *HEPH* gene and ferroportin ecoded by the solute carrier family 40 member 1 (*SLC40A1*) gene [13, 14]. Additional proteins participating in the iron uptake and non-transferrin bound iron uptake (NTBI) such as natural resistance associated protein (NRAMP2) coded by the solute carrier family 11 member 2 (*SLC11A2*) gene and zinc importer protein 14 (ZIP14) encoded by the solute carrier family 39 member 14 (*SLC39A14*) play an important role in the cellular and systemic iron metabolism together with the hemochromatosis (*HFE*) gene and protein which is connected with excessive iron loading [3, 15–20]

The role of iron in the progression and growth of tumor cells has been documented in various studies describing higher iron uptake in cancer cells due to their proliferative nature and altered metabolic needs [21–23]. The correlation between the iron content in the diet and tumorigenesis has also been proposed, suggesting iron as a risk factor for some cancer subtypes, such as haematological malignities or hepatocellular carcinoma. Molecular mechanisms underlying this phenomenon are diverse and include higher iron uptake *via* transferrin receptor, activation of hypoxia-inducible factors (HIFs) in cancer cells due to compromised function of the prolyl hydroxylases and deregulation of signaling pathways such as Wnt/β-catenin [24–31].

It has been shown that iron-deprivation is able to induce apoptosis in tumor cells, particularly in cells of hematopoietic origin. Additionally, gallium nitrate, a competitor of the iron ion, has been successfully used to treat bladder cancer in a clinical setting [32–35].

The concept of cancer stem cells (CSC) or tumor-initiating cells (TICs) has emerged recently, documenting the extreme plasticity and heterogeneity of tumor tissue. This concept states that only a small sub-fraction of tumor cells is able to initiate tumor growth *in vivo* and that cells possessing this capability cause residual disease leading to relapse and death, although it is probably not universal concept for all cancer types [36–38]. This is of crucial clinical importance and there is virtually no data on iron metabolism in these cells, with only emerging evidence that HIFs play an important role in their maintenance and renewal [39–48].

Recently, there have been several attempts to correlate iron metabolism-related genes with the survival and overall prognosis of tumor progression in breast cancer patients. Miller et al. have shown that loss of the iron excretory genes and also upregulation of the iron uptake machinery impacts the prognosis and can delineate patients that would respond well in the group of hard-to-treat individuals and *vice versa* [49]. However, changes in the expression of these genes in TICs remains elusive so far.

Our study provides an insight into iron metabolism of TICs, their response to iron withdrawal, and identifies a specific gene signature related to iron metabolism that is differentially expressed in TICs. We have also identified iron metabolism-related proteins that are differentially expressed in TICs and could be utilized in cancer diagnosis or treatment.

## RESULTS AND DISCUSSION

There are virtually no data concerning the role of iron and its metabolism in the maintenance and self-renewal of tumor-initiating cells (TICs) as yet. We thus focused our study on this particular topic and assessed iron content, sensitivity to iron chelators, iron uptake and storage, intracellular iron distribution and expression profile of iron metabolism-related genes in TICs.

### Spheres as an *in vitro* model of TICs

**Figure 1:**
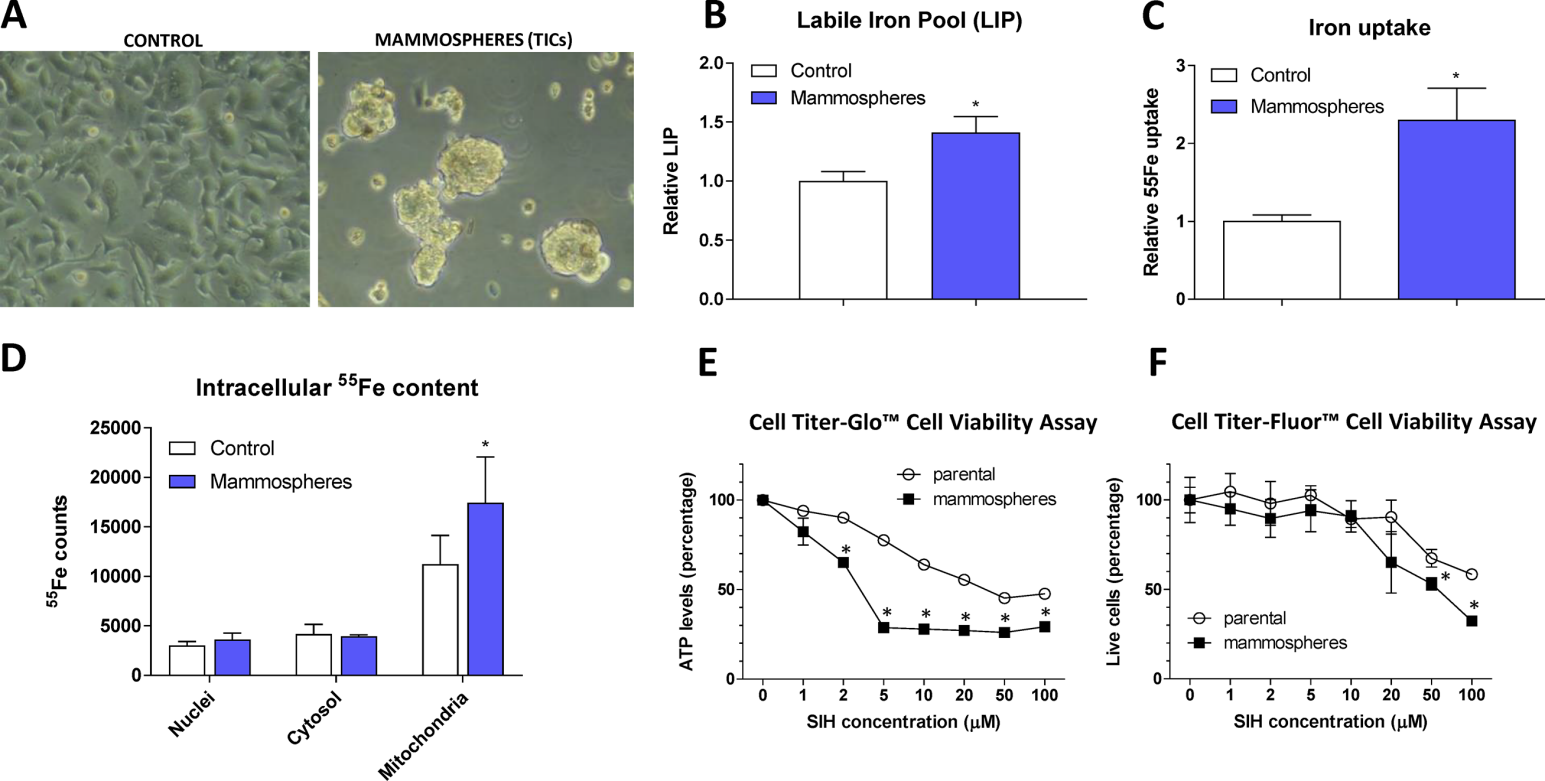
Appearance, labile iron pool, iron uptake, intracellular localization and sensitivity to chelators in tumor-initiating cells (TICs) The appearance of MCF7 cell grown either under control or sphere forming conditions is shown (**A**), together with Labile iron pool (LIP) detected by the calcein method (**B**), assessment of the ^55^Fe uptake (**C**) and intracellular distribution of ^55^Fe (**D**). Cells were also assayed for their resistance to cell death by two approaches - Cell Titer-Glow (**E**) and Cell Titer-Fluor (**F**). Experiments were performed at least in triplicate, standard error is SEM. *P*-values lower than 0.05 are denoted with a star and were calculated by the GraphPad Prism software using the unpaired *t-test*.

We have used previously published methods to generate cells growing as spheres from the breast cancer cell line MCF7 *via* two alternative methods. The first method is based on serum-free medium and cells generated by this method are referred to as “spheres” [50]. An alternative method [51] based on the non-adherent plastic resulted in cells referred to as “agar”. In our experience, the serum-free approach generated spheres with more profound expression of stem cell/epithelia-mesenchymal transition (EMT) markers, yet in some cell lines such as DU-145, only the agar approach worked as they did not form spheres under serum-free conditions. We also included a non-malignant cell line of breast origin, MCF-10A; we were unable to generate spheres from these cells by either of the above mentioned approaches, pointing to the fact that malignant but not immortalized cells are able to form spheres in our hands. The appearance of MCF7 spheres is depicted in Figure 1A and Supplementary Figure S1 shows expression of identical markers in all tested cell lines, documenting successful generation of spheres that represent *in vitro* model of TICs.

### MCF7 spheres show higher labile iron pool (LIP) and iron uptake, higher amount of iron within mitochondria and are sensitive to iron chelation

In order to characterize iron metabolism of TICs, we first inspected the level of LIP within the MCF7 sphere cells using the calcein fluorescence-based approach [52] and found significantly higher level of LIP in TICs (Figure 1B). To confirm our findings, we examined the ability of cells to acquire radioactive ^55^Fe and demonstrated that MCF7 spheres show significantly higher cellular ^55^Fe uptake (Figure 1C) and significantly higher ^55^Fe level in mitochondria (Figure 1D).

Further tests showed that application of cell permeable iron chelator such as salicyl isonicotinoyl hydrazone (SIH) resulted in decreased survival of MCF7 spheres compared to control adherent cells as measured by the Cell Titer-Glo (Figure 1E) and Cell Titer-Fluor (Figure 1F) cell viability assays. MCF7 spheres lost their ATP levels much faster and also exhibited higher numbers of dead cells compared to controls (Figure 1E–1F).

These data document higher iron uptake and labile iron pool, differential intracellular iron distribution with mitochondrial iron accumulation in MCF7 spheres as well as the necessity of iron for their survival. In order to find the underlying mechanism explaining this phenomenon, we performed expression profiling of genes that are related to iron metabolism.

### TICs derived from various cancer cell lines show deregulation of genes related to iron metabolism

We performed expression profiling of selected 40 genes that cover iron uptake, export, transport, utilization, FeS cluster biogenesis, heme metabolisms, hypoxia inducible factors and other important regulators of iron metabolism in TICs generated from MCF7, BT-474, T-47D, ZR-75–30 breast and LNCaP and DU145 prostate cancer cells. We obtained expression profiles of 34 selected iron metabolism-related genes that were detectable and showed acceptable qPCR standard curves. Raw data showing actual values and genes tested are supplied in Supplementary Table S3.

We then compared the fold change in mRNA expression between TICs prepared by the sphere approach and control cells (Supplementary Table S1) and selected genes with altered expression (> 1.5 fold change in mRNA expression) that is reproducible among cell lines (a similar change occurs in more than 60% of cell lines), resulting in the iron metabolism-related gene signature differentially expressed in TICs (*ABCB10, ACO1, CYBRD1, EPAS1, GLRX5, HEPH, HFE, IREB2, QSOX1 and TFRC)*. Individual genes participating in the iron uptake (*CYBRD1, TFRC*), iron sensing and regulation (*ACO1, IREB2*), mitochondrial iron-sulphur cluster assembly (*ABCB10, GLRX5*), hypoxia response (*EPAS1, QSOX1*), iron export (*HEPH*) and iron overload (*HFE*) are discussed below.

### Expression of cytochrome b reductase (CYBRD1) and transferrin receptor 1 (TFR1) participating in iron uptake is higher in TICs

**Figure 2:**
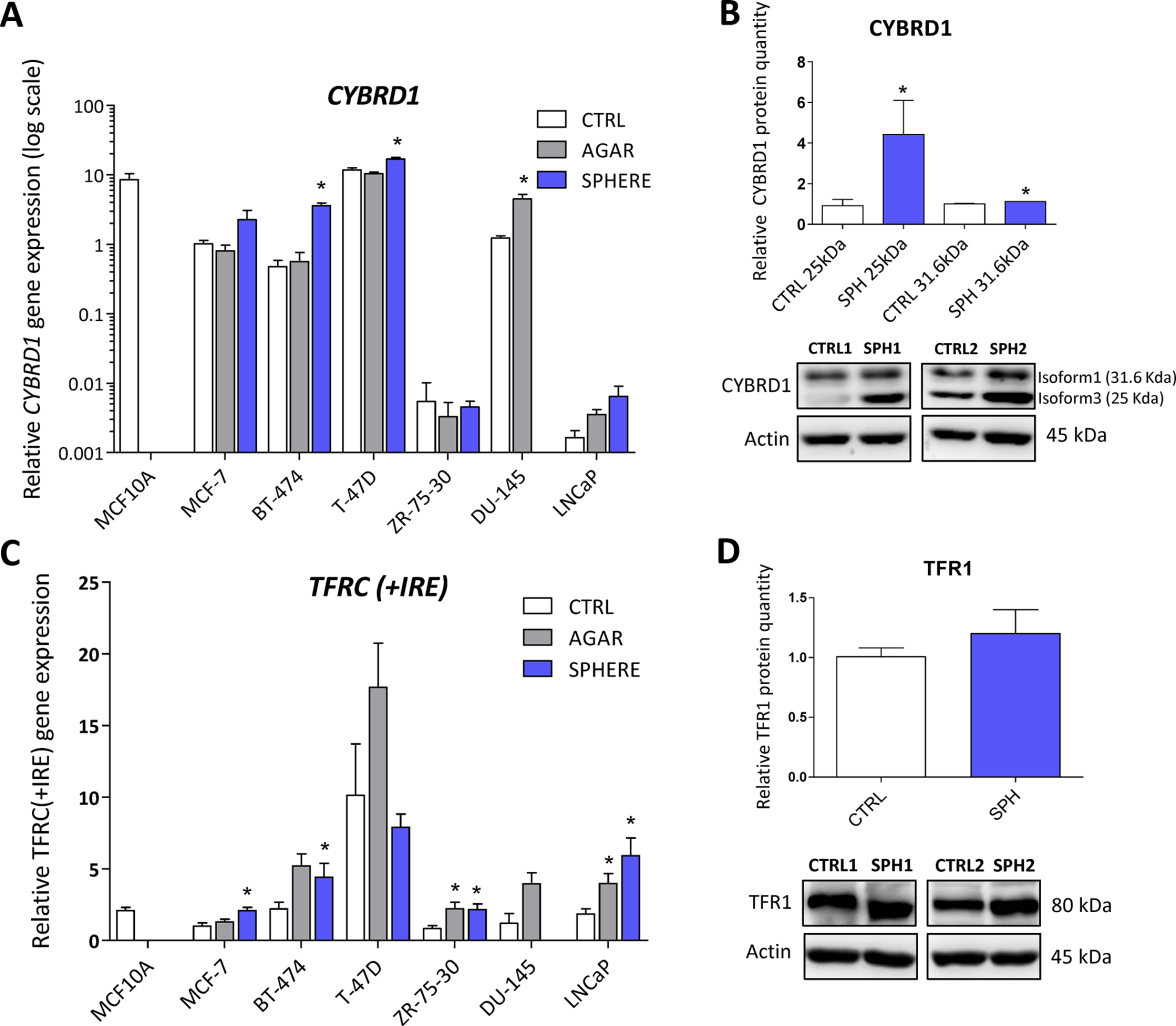
Expression of cytochrome b reductase (CYBRD1) and transferrin receptor 1 (TFR1) participating in iron uptake is higher in tumor-initiating cells (TICs) Expression of the *CYBRD1 g*ene at the mRNA level in breast non-malignant cell line MCF10A, in TICs derived from breast cancer cell lines MCF-7, BT-474, T-47D and ZR-75-30 as well as from prostate cancer cell lines DU-145 and LNCaP has been determined (**A**) together with protein levels in the MCF-7 cell line (CTRL) and MCF-7 derived spheres (SPH) (**B**). Similarly, the expression of the *TFRC* gene at the mRNA (**C**) level as well as protein level (**D**) in TICs is documented. Experiments were performed at least in triplicate, standard error is SEM, *p*-values lower than 0.05 are denoted with a star and were calculated by the GenEx software using the unpaired *t-test* and plotted with GraphPad prism software. The protein expression was quantified by the image J software from 2 to 5 independent samples, standard error is SEM, *p*-values lower than 0.05 are denoted with a star and were calculated and plotted in GraphPad prism, using the unpaired *t-test*.

CYBRD1 is an enzyme highly expressed at the duodenal brush border membrane. Product of the *CYBRD1* gene reduces ferric iron to ferrous iron and plays an important role in iron uptake from the intestine [4]. Its role in cancer is only emerging with documented overexpression in colorectal and oesophageal cancer [53, 54]. Our data showed upregulation of *CYBRD1* mRNA in most cell lines tested (MCF7, T-47D, BT-474, ZR-75–30, DU-145) with approximately 2 to 7-fold induction in TICs (Figure 2A, Supplementary Table S1), and this has been replicated on the protein level for the smaller 25 kDa CYBRD1 isoform in our MCF7 sphere model (Figure 2B). Since molecular mechanism linking CYBRD1 to cancer is missing at this point, we can only speculate that this enzyme could be regulated by members of the HIF family and enhance uptake of non-transferrin bound iron [55].

*TFRC* gene codes for TFR1 protein, a critical component of the transferrin-bound iron uptake. Elevated expression of TFR1 has been frequently reported in many cancers with possible links to poor prognosis and acceleration of the disease [27, 53, 54, 56]. The expression of this gene is reproducibly elevated in TICs derived by the sphere approach in MCF7, BT-474, LNCaP and ZR-75–30, similarly, this change is also seen with the “agar” approach in BT-474, DU-145, LNCaP, T-47D and ZR-75–30 cells (Figure 2C, Supplementary Table S1). On the protein level, we could see increased levels in spheres derived from MCF7 cells even though not reaching the statistical significance of *p* = 0.05 (Figure 2D). Since the expression of TFR1 is regulated by the iron responsive protein/iron responsive element (IRP/IRE) system, we focused on the expression and activity of its components (ACO1, IREB2).

### Iron responsive protein/Iron responsive element (IRP/IRE) components are deregulated and show activation of the IRP/IRE binding in TICs

**Figure 3:**
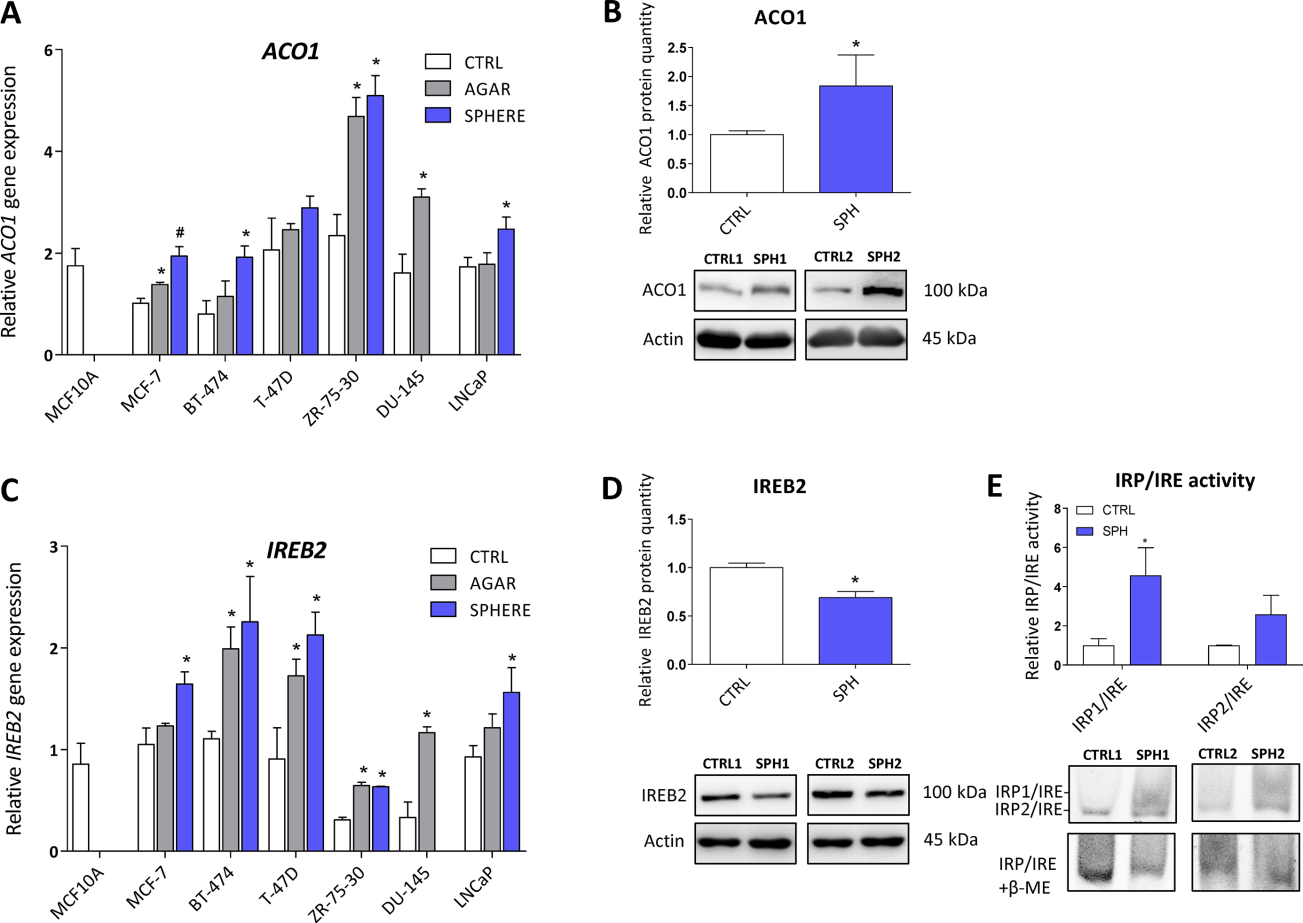
Iron responsive protein/Iron responsive element (IRP/IRE) components are deregulated and show activation of the IRP/IRE binding in tumor-initiating cells (TICs) Expression of the *ACO1 g*ene at the mRNA level in breast non-malignant cell line MCF10A, in TICs derived from breast cancer cell lines MCF-7, BT-474, T-47D and ZR-75-30 as well as from prostate cancer cell lines DU-145 and LNCaP has been determined (**A**) together with protein levels in the MCF-7 cell line (CTRL) and MCF-7 derived spheres (SPH) (**B**). Similarly, the expression of the *IREB2* gene at the mRNA (**C**) level as well as protein level (**D**) in TICs is documented. Experiments were performed at least in triplicate, standard error is SEM, *p*-values lower than 0.05 are denoted with a star and were calculated by the GenEx software using the unpaired *t-test* and plotted with GraphPad prism software. Number sign denotes statistical significance involving Dun-Bonferroni correction. Panel (**E**) illustrates the IRP/IRE activity measured by the fluorescent EMSA. The protein expression was quantified by the image J software from 2 to 5 independent samples, standard error is SEM, *p*-values lower than 0.05 are denoted with a star and were calculated and plotted in GraphPad prism, using the unpaired *t-test*.

Aconitase, also known as IRP1 or iron-responsive element-binding protein 1 (IREB1) is encoded by the *ACO1* gene. Under normal conditions, this protein functions as a metabolic enzyme participating in the conversion of citrate to isocitrate [57]. However, since ACO1 is an enzyme containing 4Fe-4S clusters as a co-factor, under iron deprivation, this enzyme dramatically changes its conformation and becomes the IRP1 with high affinity to iron-response elements (IREs) within untranslated regions (UTR) of many genes participating in the uptake and storage of iron, resulting in stabilization (3´UTR) or translation inhibition (5´UTR) of the corresponding mRNA, aiming to increase iron uptake and decrease iron storage [8, 9, 58]. There is emerging evidence about the role of ACO1 in cancer as elevated ACO1 expression suppresses tumor growth *in vivo* and its level is higher in rectal and hepatocellular carcinoma, while the opposite has been shown in leukemic cells [59–62]. Furthermore, many studies describe higher levels of isocitrate in cancer and cancer stem cells, supporting the role of ACO1 in carcinogenesis [63–66]. In our experimental system, we see approximately 2-fold increase in *ACO1* mRNA in all tested cell lines (Figure 3A, Supplementary Table S1). Moreover, these changes were replicated on the protein level of ACO1, where elevated protein levels are seen in the MCF7 sphere model (Figure 3B). Our data support the notion that ACO1 is upregulated in TICs and may play an important role in their biology.

The second protein binding to IREs is encoded by the *IREB2* gene and is also known as IRP2. This protein plays a critical role in the response to iron deprivation and is stabilized under conditions of low iron while under normal iron levels it is rapidly degraded [53, 67–69]. The role of IRP2 in carcinogenesis is only beginning to be appreciated [53, 70–73]. We have found mild, yet significant, upregulation of *IREB2* mRNA in all TICs derived by the sphere approach, with an average 2-fold change (Figure 3C, Supplementary Table S1). Contrary to this, we have detected an inverse relationship on the protein level, with downregulation of the IREB2 protein in MCF7 sphere cells (Figure 3D). This, though, has a plausible explanation, as IRP2 is normally degraded when iron level is high enough and its turnover is mediated by ubiquitinylation [74]. Since TICs contain higher levels of LIP, a decrease in IRP2 levels is expected.

We have further analysed the ability of IRP1/2 to bind the IRE motif and regulate iron uptake and storage by a modified electrophoretic mobility shift assay (EMSA). Our data clearly document activation of the IRP/IRE system showing enhanced binding mostly of the IRP1 to the IRE sequence of human ferritin (Figure 3E), which is in agreement with observed higher iron uptake, predicting that ferritin level would be decreased.

Interestingly, IRP1 activity is regulated by the absence of the assembled FeS cluster as already mentioned. We have thus focused our attention on the components of the mitochondrial FeS cluster biogenesis that show differential expression according to our expression profiling data.

### Protein levels of the ATP Binding Cassette Subfamily B Member 10 (ABCB10) and glutaredoxin 5 (GLRX5) participating in mitochondrial FeS cluster assembly are decreased in TICs

**Figure 4:**
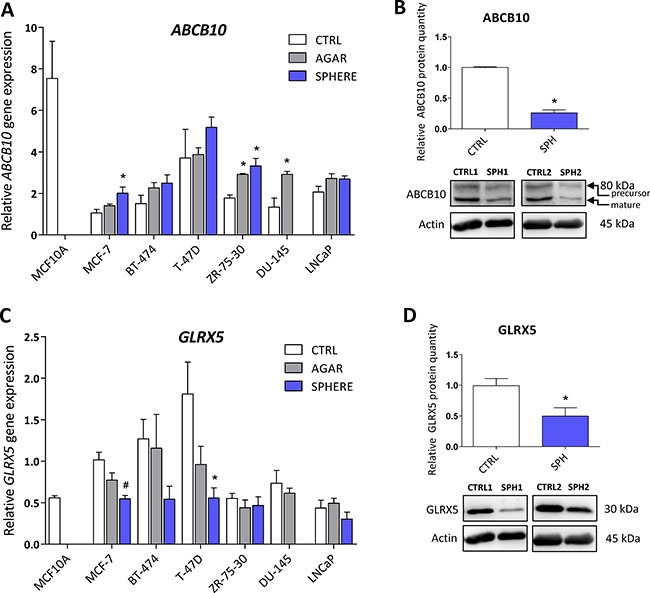
Protein levels of the ATP Binding Cassette Subfamily B Member 10 (ABCB10) and glutaredoxin 5 (GLRX5) participating in mitochondrial FeS cluster assembly are decreased in tumor-initiating cells (TICs) Expression of the *ABCB10 g*ene at the mRNA level in breast non-malignant cell line MCF10A, in TICs derived from breast cancer cell lines MCF-7, BT-474, T-47D and ZR-75-30 as well as from prostate cancer cell lines DU-145 and LNCaP has been determined (**A**) together with protein levels in the MCF-7 cell line (CTRL) and MCF-7 derived spheres (SPH) (**B**). Similarly, the expression of the *GLRX5* gene at the mRNA (**C**) level as well as protein level (**D**) in TICs is documented. Experiments were performed at least in triplicate, standard error is SEM, *p*-values lower than 0.05 are denoted with a star and were calculated by the GenEx software using the unpaired *t-test* and plotted with GraphPad prism software. Number sign denotes statistical significance involving Dun-Bonferroni correction. The protein expression was quantified by the image J software from 2 to 5 independent samples, standard error is SEM, *p*-values lower than 0.05 are denoted with a star and were calculated and plotted in GraphPad prism, using the unpaired *t-test*.

ABCB10 is a protein belonging to the ABC transporter family that has mitochondrial localization, participates in the mitochondrial FeS cluster biogenesis and was proposed to mediate protection from reactive oxygen species (ROS) [75]. Its expression has been linked to erythroid heme synthesis; *Abcb10* knockout mice suffer from anemia and show mitochondrial iron accumulation [76–78]. Even though our data document rather small (2-fold), yet significant, increase in *ABCB10* mRNA expression in all tested cell lines (Figure 4A, Supplementary Table S1), we detected a profound decrease in the ABCB10 protein level in the MCF7 sphere model of TICs (Figure 4B). This suggests that ABCB10 protein level is probably regulated by a posttranscriptional mechanism. Our data are consistent with the fact that ABCB10 expression is increased during erythroid differentiation while TICs represent de-differentiated cells with stem cell properties [79] and also comply with the fact that low level of ABCB10 is connected with mitochondrial iron accumulation as seen in Figure 1D.

Glutaredoxin 5 is another component of the mitochondrial FeS cluster machinery. This protein is an important member of the redox balance system, being able to reduce the S-S bonds into free SH groups; oxidized GLRX5 is then non-enzymatically reduced by glutathione (GSH) [80–82]. This protein is critically required for normal mitochondrial FeS cluster biogenesis as its disruption is associated with autosomal recessive pyridoxine-refractory sideroblastic anemia [6, 7, 83–85]. There is a scarce evidence about the role of GLRX5 in carcinogenesis, documenting higher expression of GLRX5 in hepatocellular carcinoma [86]. Interestingly, the expression of this critical component of FeS cluster biogenesis was supressed on the mRNA level in almost all tested cell lines (Figure 4C, Supplementary Table S1) as well as on the protein level in the MCF7 sphere model (Figure 4D). Thus, we can speculate that lower protein levels of GLRX5 may reduce proper FeS cluster biogenesis and since FeS clusters are essential for proper DNA repair and replication [1, 87], these alterations could lead to genomic instability of TICs.

### Reduced enzymatic activity of FeS cluster containing enzymes, reduced glutathione (GSH) content and increased reactive oxygen species (ROS) levels in TICs

**Figure 5:**
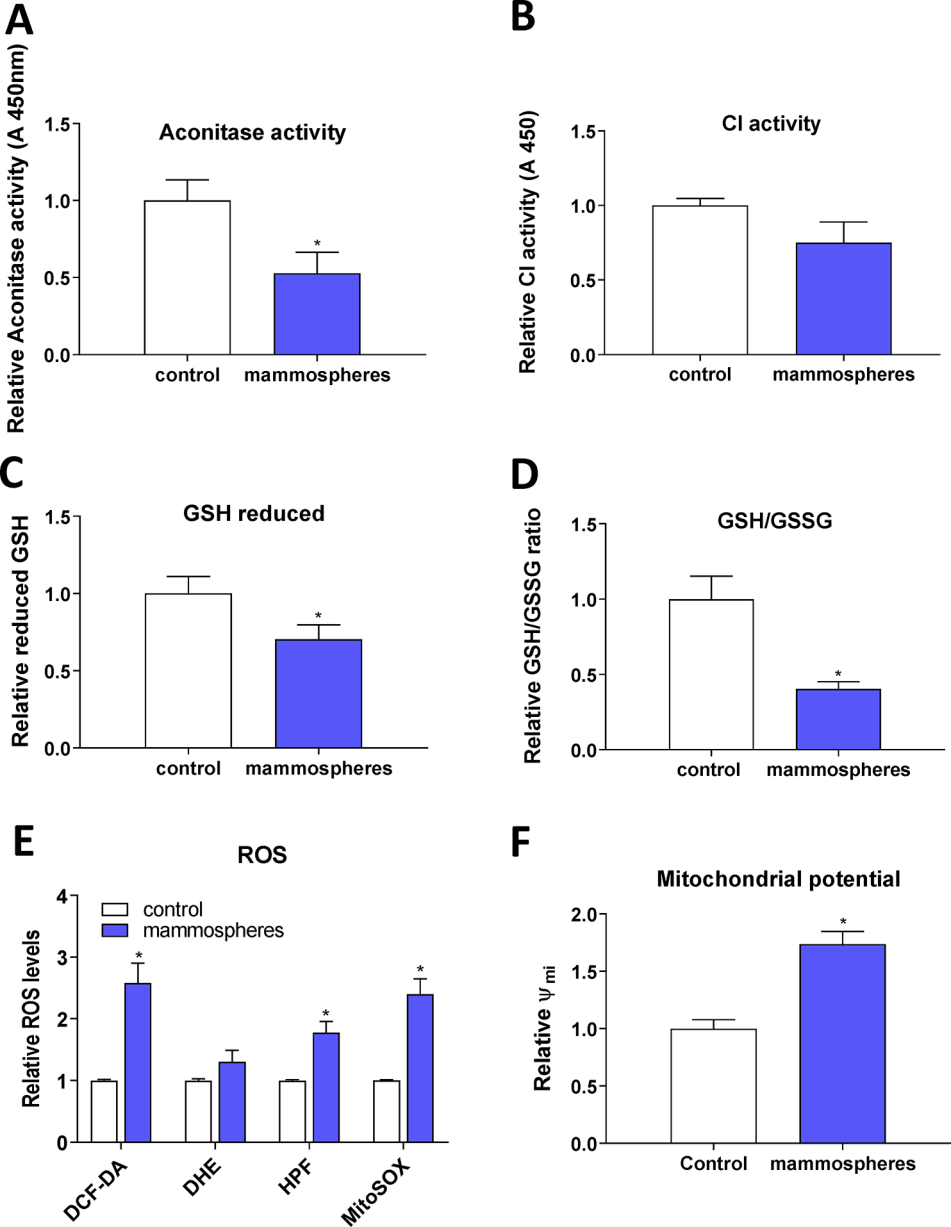
Reduced enzymatic activity of FeS cluster containing enzymes, reduced glutathione (GSH) content and increased reactive oxygen species (ROS) levels in tumor-initiating cells (TICs) Cell cultured under control conditions (control) or in sphere medium (mammospheres) were assessed for the enzymatic activity of aconitase (**A**) and mitochondrial respiratory complex I (**B**). Further tests also measured the reduced glutathione (GSH) (**C**) and the ratio of reduced/oxidized glutathione (GSH/GSSG) ratio (**D**). Spheres also show higher ROS production (**E**) measured by 2′,7′-dichlorofluorescein diacetate (DCF-DA), dihydroethidium (DHE), hydroxyphenylfluorescein (HPF) and mitochondrial superoxide indicator (mitoSOX). Spheres also show higher mitochondrial potential measured by tetramethylrhodamine methylester (TMRM) (**F**). Experiments were performed at least in triplicate, standard error is SEM, *p*-values lower than 0.05 are denoted with a star and were calculated by the GraphPad Prism software using the unpaired *t-test*.

As we detected activation of the IRP/IRE system and changes in the expression of ABCB10 and GLRX5 that may reduce proper assembly and transport of FeS clusters, we assessed the enzymatic activity of ACO1 (both the cytosolic and mitochondrial form) and also mitochondrial respiratory complex I. Both of these enzymes require FeS clusters for their enzymatic function and lack of properly formed FeS clusters should lead to their lower enzymatic activity. Indeed, our data support this scenario (Figure 5A, 5B). Furthermore, as the decreased enzymatic ACO1 activity is linked to oxidative damage [88], we tested levels of reduced GSH and the GSH/GSSG ratio that were significantly reduced in MCF7 spheres (Figure 5C, 5D). The level of ROS, assessed by 2′,7′-dichlorofluorescein diacetate (DCF-DA), hydroxyphenylfluorescein (HPF, detecting hydroxyl radical) and mitochondrial superoxide indicator (mitoSOX), was significantly higher in TICs (Figure 5E), as was mitochondrial potential measured by the tetramethylrhodamine, methyl ester (TMRM) staining (Figure 5F). Higher generation of ROS is possibly due to mitochondrial iron loading and higher labile iron pool inside the cells, supported by the fact that levels of reduced GSH and the GSH/GSSG ratio were significantly lower in TICs, pointing to higher oxidative stress. Since we detected low enzymatic activity ACO1, which is dependent on FeS clusters and inhibited by ROS [88, 89], we further examined cellular response to hypoxia, in particular the expression and activity of hypoxia inducible factors (HIFs) as these are also regulated by iron [90].

### Expression of genes related to hypoxia (Endothelial Per-ARNT-Sim Domain Protein 1, *EPAS1*), cellular quiescence and extracellular matrix remodelling (Quiescin Sulfhydryl Oxidase 1, *QSOX1*) is elevated in TICs

**Figure 6:**
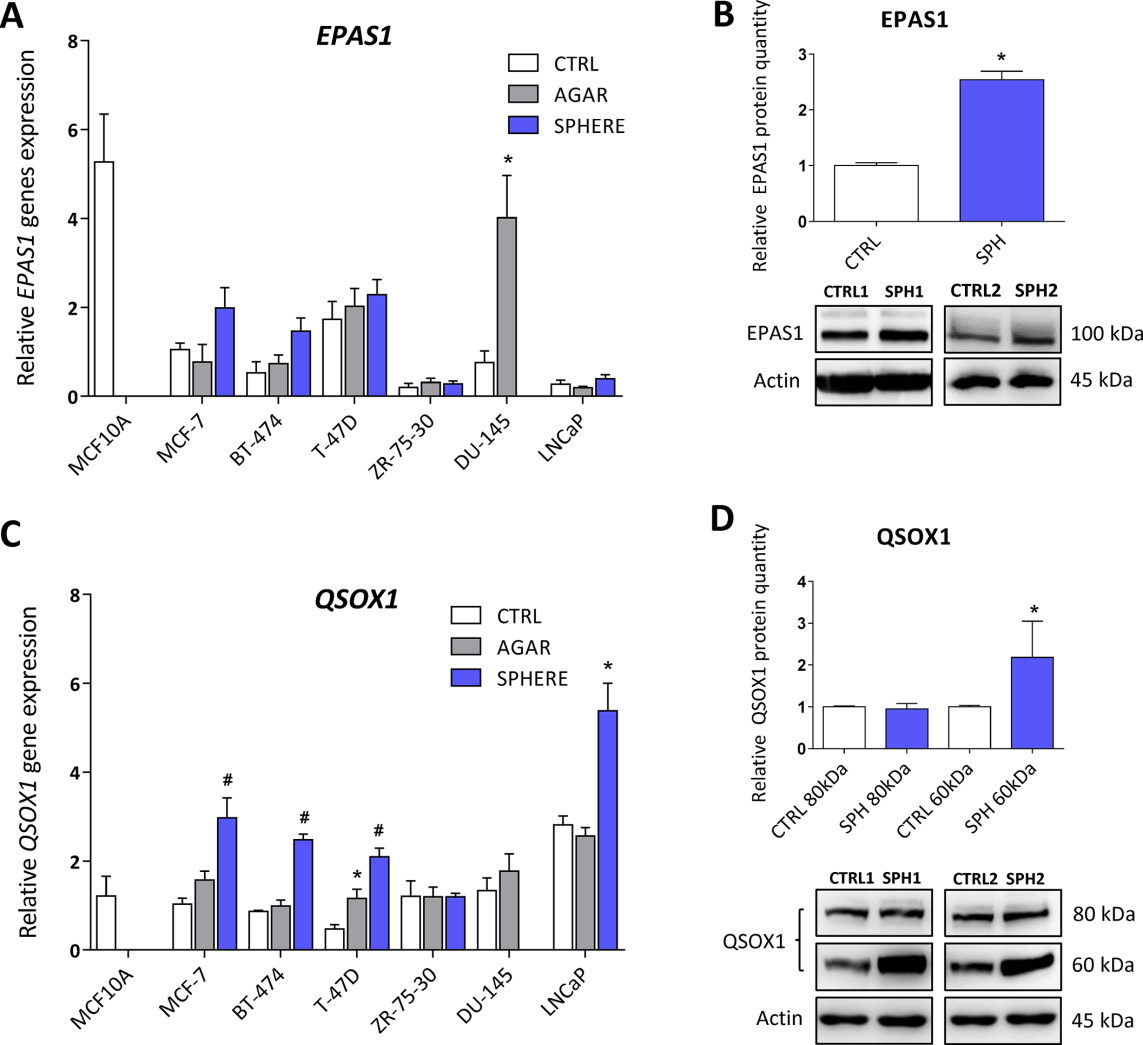
Expression of genes related to hypoxia (Endothelial PAS Domain Protein 1, EPAS1), cellular quiescence and extracellular matrix remodelling (Quiescin Sulfhydryl Oxidase 1, QSOX1) is elevated in tumor-initiating cells (TICs) Expression of the *EPAS1 g*ene at the mRNA level in breast non-malignant cell line MCF10A, in TICs derived from breast cancer cell lines MCF-7, BT-474, T-47D and ZR-75-30 as well as from prostate cancer cell lines DU-145 and LNCaP has been determined (**A**) together with protein levels in the MCF-7 cell line (CTRL) and MCF-7 derived spheres (SPH) (**B**). Similarly, the expression of the *QSOX1* gene at the mRNA (**C**) level as well as protein level (**D**) in TICs is documented. Experiments were performed at least in triplicate, standard error is SEM, *p*-values lower than 0.05 are denoted with a star and were calculated by the GenEx software using the unpaired *t-test* and plotted with GraphPad prism software. Number sign denotes statistical significance involving Dun-Bonferroni correction. The protein expression was quantified by the image J software from 2 to 5 independent samples, standard error is SEM, *p*-values lower than 0.05 are denoted with a star and were calculated and plotted in GraphPad prism, using the unpaired *t-test*.

HIFs are normally degraded by the action of prolyl hydroxylases and ubiquitinylation, resulting in proteasomal degradation. Prolyl hydroxylases require iron to carry out their action, and hypoxia inducible factor 2 (HIF2α) has been demonstrated as a direct target of ACO1 that does not contain FeS clusters and functions as an iron sensor, IRP1 [90]. Additionally, ROS have been also shown to stabilize HIF2α [91]. In accordance with higher IRP1 activity and higher ROS levels in TICs, our profiling data support the role of genes connected with hypoxic response in the phenotype of TICs, in particular the *EPAS1* gene coding for the HIF2α protein. This protein normally regulates the response of cells to hypoxia but has been also linked to the cancer stem cell phenotype [92]. The actual role of EPAS1 in carcinogenesis has been documented for paragangliomas, pheochromocytomas, bladder cancer, ovarian cancer, and a link to the oxidative phosphorylation and stem cell features has been suggested [24, 40, 93–100]. Interestingly, its expression at the mRNA level is upregulated in spheres derived from MCF7, BT-474, DU-145 and LNCaP spheres, although only DU-145 reached statistical significance (Figure 6A, Supplementary Table S1). These changes were then subsequently confirmed on protein level in MCF7 sphere model (Figure 6B). Thus, HIF2α could be responsible for metabolic adaptations of TICs and might also be responsible for the observed upregulation of several iron metabolism-related genes such as *CYBRD1 or QSOX1* [11, 12].

As pointed above, our analysis also revealed increase in the *QSOX1* expression. QSOX1 is a HIF target gene, connected to cellular quiescence and extracellular matrix remodelling [12]. Physiological role of this enzyme includes generation of disulphide bonds accompanied by generation of hydrogen peroxide and it contains the essential for respiratory and vegetative growth (ERV) domain homologous to the yeast Erv1p that is required for maturation of the cytosolic FeS clusters [101–105]. QSOX1 expression is induced in fibroblasts entering quiescence, and its role in cancer is beginning to appear [106] with reports stating that QSOX1 may be a specific marker for the luminal B subtype of breast cancer [107–109]. Its role has been suggested also for pancreatic and lung cancer, and for neuroblastoma [12, 110–112]. QSOX1 can also be involved in inhibition of autophagic flux in cancer cells [113]. Its role in remodelling of extracellular matrix, cell invasion and motility has also been described [114]. The *QSOX1* mRNA expression is reproducibly and significantly elevated in spheres derived from MCF7, BT-474, T-47D and also in LNCaP cells (Figure 6C, Supplementary Table S1). The increase in QSOX1 expression was confirmed on the protein level as well (Figure 6D). It is of interest that the highly induced isoform is the 66 kDa variant of this protein with a thus far elusive function. Given the fact that higher expression of QSOX1 may participate in extracellular matrix remodelling and in modulating the ratio between free SH groups and S-S bonds as well as being recognized as an HIF target, we can speculate that QSOX1 may be an important regulator of TICs maintenance and also their migration.

### Iron export machinery-related hephaestin *(HEPH)* is elevated at the mRNA and protein level while the hemochromatosis gene (*HFE)* related to systemic iron loading is increased only at the mRNA level in TICs

**Figure 7:**
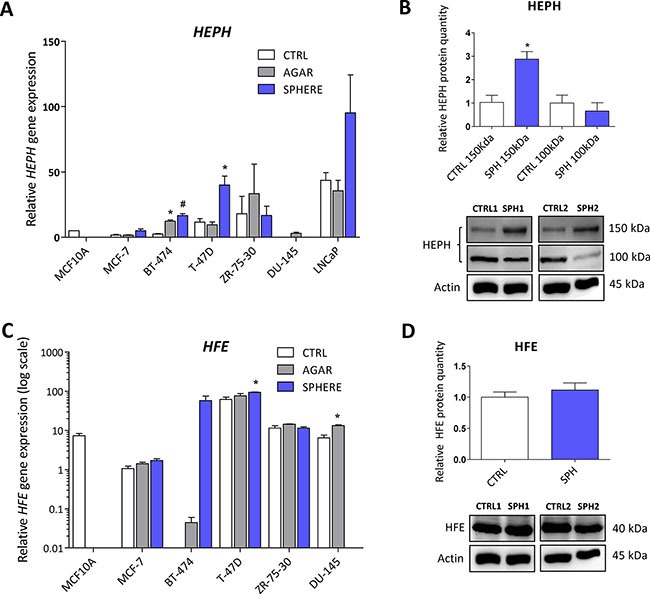
Iron export machinery-related hephaestin (HEPH) and the hemochromatosis gene (HFE) related to systemic iron loading are elevated at the mRNA level but not on the protein level in tumor-initiating cells (TICs) Expression of the *HEPH g*ene at the mRNA level in breast non-malignant cell line MCF10A, in TICs derived from breast cancer cell lines MCF-7, BT-474, T-47D and ZR-75-30 as well as from prostate cancer cell lines DU-145 and LNCaP has been determined (**A**) together with protein levels in the MCF-7 cell line (CTRL) and MCF-7 derived spheres (SPH) (**B**). Similarly, the expression of the *HFE* gene at the mRNA (**C**) level as well as protein level (**D**) in TICs is documented. Experiments were performed at least in triplicate, standard error is SEM, *p*-values lower than 0.05 are denoted with a star and were calculated by the GenEx software using the unpaired *t-test* and plotted with GraphPad prism software. Number sign denotes statistical significance involving Dun-Bonferroni correction. The protein expression was quantified by the image J software from 2 to 5 independent samples, standard error is SEM, *p*-values lower than 0.05 are denoted with a star and were calculated and plotted in GraphPad prism, using the unpaired *t-test*.

We have also detected changes in the expression of the *HEPH* gene, coding for the multi copper oxidase hephaestin, which physiologically helps iron transport from enterocytes [14, 115–117]. Disruption of the *HEPH* gene results in hypochromic microcytic anemia and retinal iron overload [116, 118, 119]. In relation to cancer, HEPH expression was reduced in colorectal carcinoma and loss of HEPH was associated with more advanced disease [53]. In our sphere model of TICs, the expression of *HEPH* mRNA was significantly higher in BT-474 and T-47D breast cancer cells while other cell lines also showed increased expression but did not reach statistical significance (Figure 7A, Supplementary Table S1). The HEPH protein level showed a significant increase in HEPH protein isoform at 150 kDa, while the 100 kDa isoform is decreased in the MCF7 sphere model (Figure 7B). Thus, HEPH role in TICs is probably specific for a particular cell type and individual isoforms and their response to iron and involvement in carcinogenesis might differ.

Interestingly, the *HFE* gene coding for the hemochromatosis protein was also altered in TICs. This gene and its C282Y mutant form have long been connected to excessive iron loading in hemochromatic patients. The accumulation of iron caused by mutation in the *HFE* gene increases the risk of cancer development [53, 120–122]. Unexpectedly, we detected an increase in *HFE* mRNA level in TICs generated by the sphere approach in MCF7, BT-474, T-47D and DU-145 cells (Figure 7C, Supplementary Table S1). However, on the protein level, no changes were seen in MCF7 sphere model (Figure 7D). Thus, although there seems to be a significant upregulation of *HFE* mRNA, the lack of response on the protein levels rather suggests that the HFE protein is not linked to the TICs phenotype.

To gain further insight into iron metabolism of TICs, we have assessed the expression of additional important regulators of iron uptake, transfer, storage and export that are known to be regulated on the level of protein rather than on the level of transcription. Since these proteins are regulated on the protein levels they were not picked up by the expression profiling.

### Protein levels of regulators related to iron transport (NRAMP2, Natural resistance associated macrophage protein 2) and iron storage (ferritin) are decreased while expresssion of proteins participating in non transferrin bound iron (NTBI) uptake (ZIP14, Zinc importer protein 14) and iron export (ferroportin) does not differ in TICs

**Figure 8:**
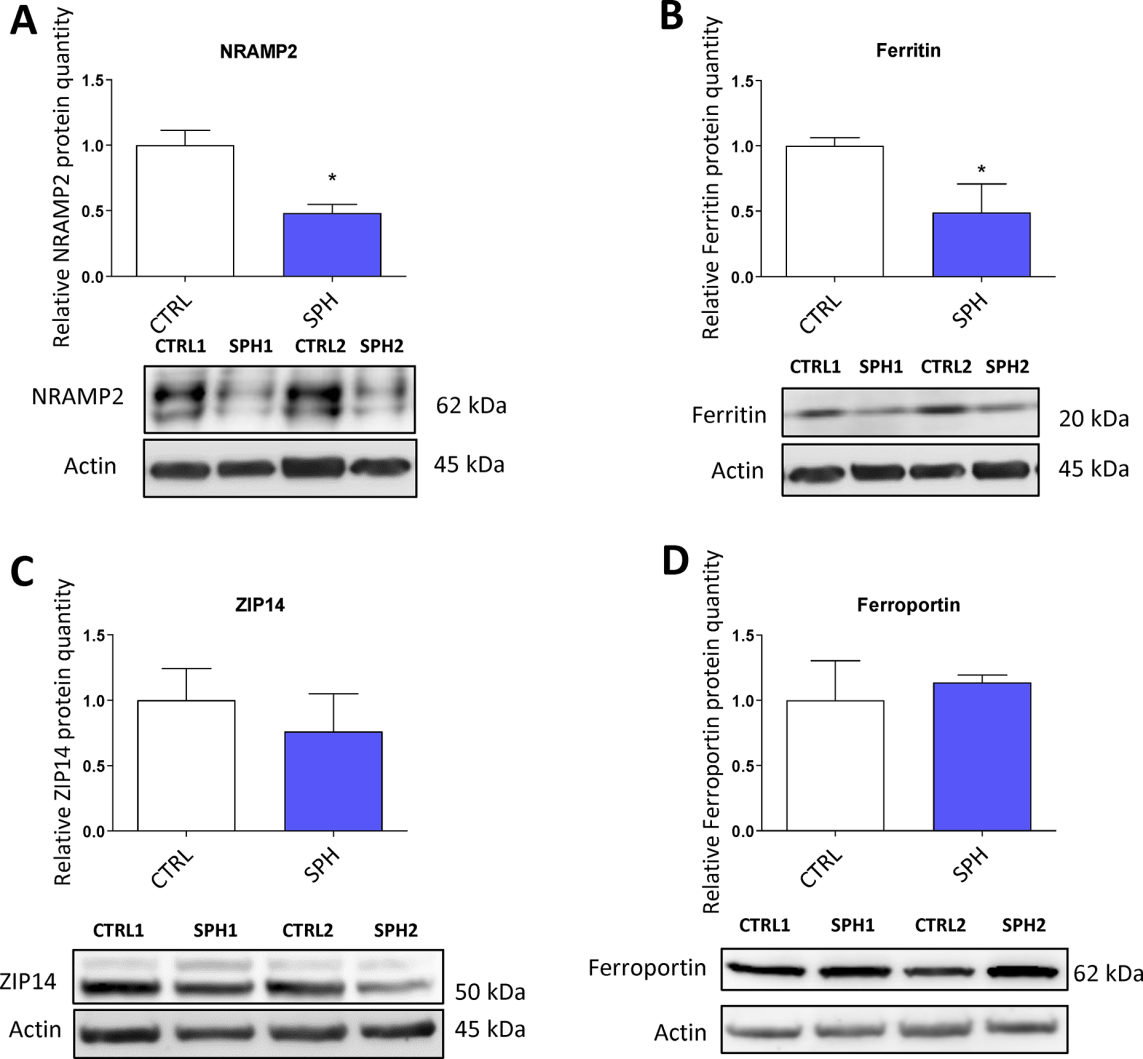
Protein levels of regulators related to iron transport (NRAMP2, Natural resistance associated macrophage protein 2) and iron storage (ferritin) are decreased while expression of proteins participating in non transferrin bound iron (NTBI) uptake (ZIP14, Zinc importer protein 14) and iron export (ferroportin) does not differ in tumor-initiating cells (TICs) Protein levels of NRAMP2 (**A**), ferritin (**B**), zinc transporter protein 14 (**C**) and ferroportin (**D**) in the MCF-7 cell line (CTRL) and MCF-7 derived spheres (SPH) are shown. The protein expression was quantified by the image J software from 2 to 5 independent samples, standard error is SEM, *p*-values lower than 0.05 are denoted with a star and were calculated and plotted in GraphPad prism, using the unpaired *t-test*.

First, we examined the expression of solute carrier family 11 member 2 (*SLC11A2*) coding for a protein known as NRAMP2 or DMT1 (Divalent metal transporter 1). NRAMP2 is known to transport iron from the gut lumen into enterocytes and also participates in the release of transferrin iron from the acidic environment of the lysosomes into cytosol, other studies also suggest its role in the non-transferrin bound iron (NTBI) uptake [16, 123, 124]. Its role in carcinogenesis is not well described with possible role in colorectal and oesophageal cancer [54, 61, 125]. Interestingly, the level of *SLC11A2* mRNA containing a functional IRE motif (+IRE) measured by the gene profiling, did not dramatically change with exception of the DU-145 cell line; however, the non-IRE variant mRNA seemed significantly upregulated in most cell lines (Supplementary Figure S3A). Yet, NRAMP2 protein levels were significantly reduced in MCF7 sphere model (Figure 8A). This could be an explanation why there is paradoxically higher LIP while cells show activation of IRP/IRE system, since lack of NRAMP2 would leave the acquired iron locked in the lysosomes, unavailable to be incorporated into the active sites of enzymes and proteins.

Further, we analyzed levels of ferritin at mRNA and protein levels (encoded by *FTH* and *FTL*) as ferritin is a major iron storage protein that is capable of binding tremendous amount of iron, storing it and then releasing it when needed. Ferritin is encoded by *FTH1* responsible mainly for the ferroxidase activity and also *FTL1* which plays a role in iron nucleation and protein stability [5, 126]. Ferritin has been linked to progression of breast, ovarian, pancreatic and prostate cancer [28, 127–129]. In our data set, there were almost no changes in *FTH* and a slight increase in *FTL* mRNA level only in MCF7 and BT-474 (Supplementary Figure S3B), while the protein levels were markedly reduced (Figure 8B). This is in agreement with the activation of the IRP/IRE system implicating that iron storage is reduced and provides another independent validation that the IRP/IRE system activity is increased. Also a reduction of the ferritin levels is in line with a current study suggesting that FTH is a negative regulator of ovarian stem cell expansion [129]

We also analysed expression of an important player in the non-transferrin bound iron (NTBI), the ZIP14 protein encoded by the *SLC39A14* gene which has been shown to transport iron [18, 19, 130]. Its role in carcinogenesis is so far unclear with possible role in hepatocellular cancer [131, 132] Interestingly, on the mRNA level we were able to detect an upregulation in most cell lines tested (Supplementary Figure S3C), yet, on the protein level, we did not detect any significant change in by the MCF7 sphere model (Figure 8C) suggesting that the detected 52 kDa form of ZIP14 is probably not participating in higher iron uptake in TICs.

Additionally, we have examined the expression of the solely know iron exporter, ferroportin, (FPN) encoded by the *SLC40A1* gene [15, 67, 133, 134]. A report connecting lower expression of ferroportin to iron accumulation and cancer progression exists but other research on this topic remain scarce [49]. Our data documented no significant change in the mRNA level in most of the cell lines tested with exception of BT-474 and T-47D where it was increased (Supplementary Figure S3D). However, western blot analysis of its protein level did not show any significant change in MCF7 sphere model (Figure 8D). This suggests that a change in iron export is not the underlying cause of higher LIP in TICs and the observed changes are rather related to iron uptake and may reflect higher level of improperly assembled FeS clusters.

### Expression profiling and protein analysis of the tamoxifen resistant (TAMR) MCF7 cells representing an alternative model of TICs

To further validate our findings on another *in vitro* model, we used the tamoxifen resistant MCF7 cells (TAMR) that exhibit features of stem cells (Supplementary Figure S5A) in agreement with published literature [135–137].

These cells mostly recapitulated findings seen in TICs represented by the MCF7 spheres. They show higher levels of LIP, higher ROS, reduced GSH (data not shown) and identical regulation of ABCB10, ACO1, GLRX5, EPAS1, 100kDa isoform of HEPH, IREB2, SLC39A14, SLC40A1 and QSOX1 with statistically significant changes on the mRNA and protein level (Supplementary Figure S5B, Supplementary Figure S6) pointing to high similarity in terms of iron gene signature. Yet, we have detected different regulation in protein levels of 150 kDa isoform of HEPH, TFRC, Ferritin and SLC11A2 (Supplementary Figure S6). These differences might be related to the presence of tamoxifen, an agent that is known to generate ROS, resulting in adaptation to permanent oxidative stress. One of the possible mechanism might be upregulation of ferritin which is able to inactivate free iron by its ferroxidase activity [138].

Furthermore, unlike MCF7 spheres, TAMR cells show higher SLC11A2 (DMT1) levels and no change in TFR1 levels. Yet, it might just be a different mechanism how to acquire iron, which seem to be more dependent on TFR1 in spheres, while TAMR cells probably use more of the non-transferrin bound iron *via* the SLC11A2, both converging on higher iron uptake as suggested by Miller et al [49]. Thus, the alternative model of TAMR cells confirmed majority of changes seen in the model of MCF7 spheres and supports the importance of iron in the biology of TICs.

### Analysis of the iron metabolism-related genes in leukemia-initiating cells (LICs) from leukemic mice *in vivo*

To further expand our findings from an *in vitro* conditions to an *in vivo* conditions, we used a murine model of acute promyelocytic leukemia (APL) that allows for identification and isolation of LICs, based on the depletion cocktail-, c-kit+, and CD34+ expression profile. Non-LICs from leukemic mice were characterized by depletion cocktail-, c-kit-, and CD34- expression profile and the expression of lactoferrin (Ltf) that is highly induced in the differentiated cells [139]. Unfortunately, we were not able to determine expression on the level of protein since the number of cells that are obtained after sorting is very low for some sorted populations and western blot of several proteins is technically unfeasible.

We detected statistically significant upregulation of *Glrx5* and *Tfrc* mRNA between leukemic CD34-/c-kit- (non-LICs) and CD34+/c-kit+ (LICs) populations as well as significant differences in the expression of *Cybrd1* and *Qsox1* between CD34+/c-kit+ (LICs) populations of normal and leukemic mice (Supplementary Figure S2A, Supplementary Table S2). Furthermore, expression of *Aco1, Epas1, Glrx5, Hfe, Ireb2 and Tfrc* mRNA was significantly altered between the CD34-/c-kit- (non-LICs) populations of normal and leukemic cells (Supplementary Figure S2A, Supplementary Table S2). Comparing the sorted CD34+/c-kit+(LICs) population to whole bone marrow, we could see a similar picture, which is statistically significant upregulation in *Epas1* and *Glrx5* and changes in the *Abcb10* and *Qsox1* expression that were very close to significance (Supplementary Figure S2B, Supplementary Table S2). Thus, we did see significant changes in expression of iron metabolism-related genes supporting the evidence that iron uptake in these cells is higher and their FeS cluster metabolism and hypoxia related genes show higher expression.

### Principal component analysis (PCA) based on the expression of selected iron-metabolism related genes is able to distinguish tumor-initiating cells (TICs) *in vitro* and leukemia-initiating cells (LICs) in the acute promyelocytic leukemia (APL) mouse model

**Figure 9:**
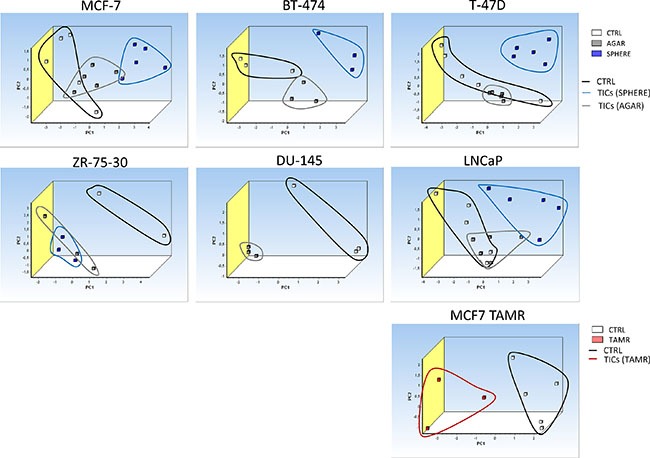
Principal component analysis (PCA) discriminates tumor-initiating cells (TICs) from cancer cells based on gene expression of the selected iron metabolism-related genes (Aco1, Abcb10, Cybrd1, Epas1, Glrx5, Heph, Hfe, Ireb2, Qsox1 and Tfrc) Principal component analysis (PCA) based on selected iron metabolism-related genes was run on malignant breast MCF-7, BT-474, T-47D, ZR-75-30 and malignant prostate DU-145 as well as LNCaP cell lines using the GenEx software which was also used for plotting the PCA. White squares depict control conditions, grey show agar conditions, blue boxes show sphere conditions and red boxes show TAMR cells. Individual clusters were also highlighted with corresponding lines using identical colors.

In order to define whether the expression of the identified differential iron metabolism-related gene signature is able to distinguish TICs from non-TICs, we performed the PCA analysis based on the expression of gene signature identified by the expression profiling consisting of *Aco1, Abcb10, Cybrd1, Epas1, Glrx5, Heph, Hfe, Ireb2, Qsox1, Tfrc* genes. The PCA clearly documents that sphere samples are clustered separately from the control adherent cancer cells in all of the tested cell lines (Figure 9). This is further documented for the TAMR cells as well (Figure 9). Furthermore, the PCA analysis with similar gene set (excluding Heph), applied on an *in vivo* APL mouse model of leukemia-initiating cells is also able to easily distinguish the population using similar gene set with exclusion of *Heph* is also able to easily distinguish the population of LICs (CD34+/c-kit+) from non-LICs (CD34-/c-kit-), thus replicating our findings in cell lines (Supplementary Figure S4). Thus, Thus, although we obtained we obtained our iron metabolism related gene signature in breast cancer TICs, and we applied it to a leukemia cells, we were clearly able to distinguish the LICs from non-LICs as well.

### Summary

In this report, we examined the role of iron in the biology of TICs. We show that TICs exhibit marked alterations in the iron metabolism and handling and also show differential gene expression of the iron metabolism-related genes, thus pointing to the importance of iron in the biology of TICs.

To gain a more detailed knowledge about the iron metabolism in TICs, we assessed many aspects related to iron metabolism. We show that TICs contain higher labile iron pool (LIP), in other words these cells contain more loosely bound iron. Free iron is known to generate ROS via the Fenton and Haber-Weiss reactions. Thus, in agreement with higher LIP in TICs, we detected higher level of hydroxyl radicals and also higher level of mitochondrial superoxide, in line with the observed accumulation of iron in mitochondria.

Since oxidative stress and labile iron pool are known to regulate the function of the iron responsive proteins (IRPs), we assessed their expression and function. Our data show that especially IRP1 known also as ACO1 is activated and binds to its cognate element, IRE. This results in higher iron uptake via TFRC and CYBRD1 and lower levels of ferritin, the major intracellular iron storage protein. As ACO1 requires FeS clusters for its enzymatic function and its IRP1 function is connected with the absence of FeS clusters, we looked into the expression of the FeS cluster assembly components. Our data clearly show that there is low expression of ABCB10 and GLRX5 proteins, both of which are located in mitochondria. Together with low amount of GSH which is required for normal FeS cluster biosynthesis, our data suggest that FeS cluster assembly is inefficient in TICs, resulting in an increase of IRP1 activity of ACO1 and possibly leading to iron accumulation in mitochondria due to low ABCB10 levels. This is further supported by lower enzymatic activity of proteins requiring FeS clusters for their catalytic activity such as mitochondrial respiratory complex I and the above mentioned ACO1. Furthermore, low levels of properly assembled FeS clusters may also underlie higher sensitivity of TICs to iron withdrawal that was observed and predispose them for genetic instability and very high plasticity.

**Figure 10:**
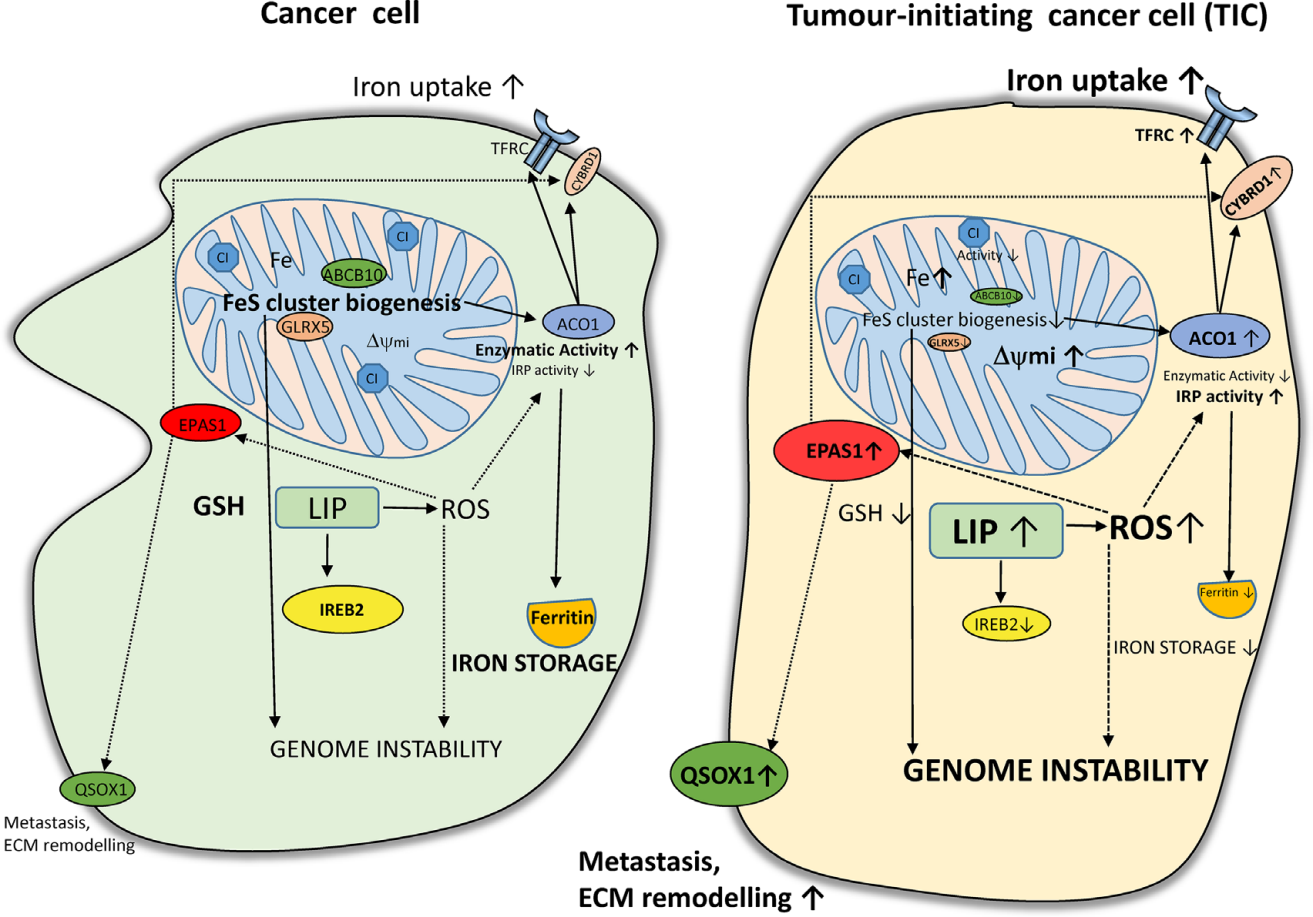
Putative scheme depicting changes in iron metabolism of tumor-initiating cells (TICs) TICs show higher levels of labile iron pool (LIP) and subsequently higher reactive oxygen species (ROS) which in connection with lower Fes cluster biogenesis (lower expression of ABCB10 and GLRX5) leading to accumulation of iron in mitochondria and possibly affecting genome stability and increasing plasticity of these cells. Alterations in the FeS cluster assembly lead to reduced activity of ACO1/IRP1 as well as mitochondrial respiratory complex I. The IRP binding activity of ACO1 is elevated resulting in higher TFRC and lower ferritin levels. Furthermore, TICs show an increase in the protein level of of EPAS1/ HIF2A connected with higher iron uptake (CYBRD1, TFRC) as well as with the extracellular matrix remodelling and redox balance equilibrium *via* the expression of QSOX1. On the other hand, reduced glutathione (GSH) is lower in TICs and these cells seem to be in a more pro-oxidative state and contain lower protein levels of IREB2/IRP2 likely reflecting increased LIP.

Interestingly, ACO1 that does not contain FeS clusters shows high IRP1 activity and stabilizes the *EPAS1* mRNA coding for the HIF2α [90]. In addition, protein stability of HIF2α is enhanced by ROS [91]. In agreement with this, we have detected higher levels of HIF2α in TICs, possibly connecting it with activation of HIF targets (DCYTB1, QSOX1). The activation of QSOX1 which is connected with extracellular remodelling and cellular quiescence might be an important step regulating cellular migration and invasion. All the above mentioned changes are depicted in summarizing Figure 10.

Other iron metabolism related proteins participating in iron export (HEPH, ferroportin), iron trafficking and non-transferrin-bound iron (NRAMP2, ZIP14) and iron storage (ferritin) were examined. We detected higher levels of the *HEPH* mRNA and higher levels of HEPH 150Kda variant, no changes in ferroportin and ZIP14 while there is a clear downregulation of ferritin and NRAMP2. Thus, in agreement with the activation of the IRP/IRE system, ferritin level is suppressed and the involvement of NRAMP2 and ZIP14 in the acquisition of iron in TICs is unlikely.

Lastly, our work identified a specific iron metabolism-related gene signature differentially expressed in TICs (*ABCB10, ACO1, CYBRD1, EPAS1, GLRX5, HEPH, HFE, IREB2, QSOX1* and *TFRC)*. The principal component analysis based on this signature is able to distinguish not only TICs *in vitro* (TICs growing as spheres and also TICs represented by the tamoxifen resistant MCF7 cells) but also LICs *in vivo*, thus confirming the importance of iron metabolism in their phenotype. Further research focusing on the role of the differentially expressed iron metabolism-related proteins in TICs biology and cancer resistance is warranted and may be of clinical importance. Studies examining the role of combination therapy using anti TICs drugs in combination with iron chelators might pave the path to a novel treatments in the future.

## MATERIALS AND METHODS

### Tissue culture and sphere generation

Cells were routinely cultivated in Dulbecco's Modified Eagle Medium (DMEM) (BT-474, DU-145, MCF7, T-47D, ZR-75–30 cells) or Roswell Park Memorial Institute medium (RPMI1640) (LNCaP cells) supplemented with 10% fetal bovine serum (FBS), 10 mM 4-(2-hydroxyethyl)-1-piperazineethanesulfonic acid (HEPES) and 2 mM glutamine. MCF-10A cells were cultivated in DMEM/F12 with 5% horse serum and antibiotics, supplemented with 0.1 ng/ml cholera toxin, 20 ng/ml epidermal growth factor (EGF), 0.5 μg/ml hydrocortisone, 1 mg/ml insulin. All cells were obtained either directly from ATCC or from prof. Lopez (Griffith University, Australia). For the generation of spheres, advanced DMEM/F12 or RPMI1640 medium supplemented with 5% of proliferation supplement, 20 ng/ml EGF, 5 ng/ml fibroblast growth factor (FGF), 4 μg/ml heparin, 10 mM HEPES, 1 mM glutamine and penicillin/streptomycin antibiotics was used. Control medium contained 5% FBS instead of proliferation supplement and was supplemented with 1 mM glutamine, 10 mM HEPES and penicillin/streptomycin. Cells generated by the agar approach were cultivated in normal serum containing medium but on a plastic ware coated with 1% agarose.

### RNA isolation and quality determination

RNA was isolated by the method using RNAzol RT according to manufacturer's instructions. Briefly, cells were collected, spun and lysed in 500 μl of RNAzol. The lysate was then mixed with 200 μl of RNAse-free water, vortexed and incubated for 10 min at room temperature (RT), and spun at 12,500 × g for 15 min. The supernatant was then mixed with 4-bromoanisole, incubated for 10 min and spun at 12,500 × g for 10 min. The supernatant was next precipitated with equal volume of isopropanol, spun at 4°C at 14,000 × g for 15 min, washed twice with 80% ethanol, dried and dissolved in RNAse free water (20 μl). RNA quantity was measured with the Nanodrop spectrophotometer (ND-1000, Thermo Scientific), and each RNA integrity was measured with the Agilent 2100 Bionalyser (Agilent Technologies).

### cDNA synthesis and sample preparation for the Fluidigm qPCR

RNA quality of the used samples was determined by the RNA integrity number score (RIN between 8–10), and cDNA was reverse-transcribed by the Maxima H minus reverse transcriptase kit (Thermo Scientific) using 400 ng total RNA as a template and oligo-dT as primers, according to manufacturer's instructions.

### Fluidigm qPCR

Primer design was performed with Primer BLAST. All assays were designed to span at least one intron and/or to have one primer covering an exon/exon boundary. Each sample was pre-amplified with mix of all primer pairs for 18 cycles. The reaction contained 5 μl of iQ Supermix (Bio-Rad), 2 μl of diluted cDNA, 1.25 μl of pre-amplification primer mix in final concentration 25 nM and 1.25 μl of water. Temperature profile was 95°C for 60 s and 18 cycles of 95°C for 15 s and 4 min at 60°C. qPCR was performed using the high-throughput platform BioMark HD System (Fluidigm) with 96.96 Dynamic ArrayIFC for gene expression. 5 μL of sample pre-mix contained 1 μl of 20x diluted preamplified cDNA, 2.5 μl of SsoFast EvaGreen Supermix (Bio-Rad), 0.25 μl of 20x SG sample loading reagent (Fluidigm) and 1.25 μl of water. Five μl of assay pre-mix contained 2 μl of 10 μM primer/probe assays, 2.5 μl of 2× assay loading reagent (Fluidigm) and 0.5 μl of water. Thermal conditions for qPCR were: 98°C for 3 min, 35 cycles of 98°C for 5 s and 60°C for 5 s. Raw data were subtracted from the gDNA control and efficiencies of individual assays were calculated from the serial dilutions of a mixed cDNA sample. Assays with insufficient efficacy or very high Cq values (> 25) were excluded from the analysis. The actual analysis was done via the GenEx software version 6, and the missing values were replaced by the mean of average value calculated from the whole group. Reference genes for normalization were identified by Normfinder; data were normalized to several reference genes *(GAPDH*, *POLR2A*, *RPLP0*, *HPRT1* and *TBP)*. The data were assessed for statistical analysis by using the unpaired *t-test* via GenEx, with the *p-value* < 0.05 was considered statistically significant and results with the Dun-Bonferroni correction are presented as well.

### Western blot analysis

Protein expression was assessed by a standard western blot assay. Briefly, cells were washed with phosphate-buffered saline (PBS) and lysed directly on a Petri dish in 1x cell lysis buffer supplemented with protease and phosphatase inhibitors. Protein concentration was measured via the bicinchinonic acid (BCA) method, and 50–80 μg of total protein was loaded in each well of the SDS-PAGE gel. Gels were then separated according to standard procedure at 20 mA per well, washed in 1× towbin buffer and blotted onto a nitrocellulose membrane via Xcell blotting module (Invitrogen) at constant voltage (35 V) for 2 h. Membranes were then blocked with 5% non-fat milk for 1 h, washed and incubated in 5% bovine serum albumin (BSA)/Tris-buffered saline (TBS)/0,1% Tween-20 with primary antibodies against ABCB10 (ThermoScientific #PA5-30468, dil. 1:1000), ACO1 (ThermoScientific #PA5-27824, dil. 1:1000), CYBRD1 (Bioss #bs-8297R, dil. 1:1000), EPAS1 (ThermoScientific #PA116510, dil. 1:1000), GLRX5 (Bioss #bs-13395R, dil. 1:1000), HEPH (Bioss #bs-15458R, dil. 1:1000), HFE (Bioss #bs-12335R, dil. 1:1000), IREB2 (ThermoScientific #PA116544, 1:500), QSOX1 (Sigma #SAB2700031, dil. 1:1000), TFRC (ThermoScientific #13-6800, dil. 1:2500), SLC39A14 (Abcam #ab191199, dil. 1:2000), SLC40A1 (Bioss #bs-4906R, dil. 1:1000) SLC11A2 (Cell Signalling #15083, dil. 1:1000), Ferritin (Abcam #ab75973, dil. 1:1000), Actin (Thermoscientific #MA5-15739-HRP, dil. 1:2000), Tubulin (Abcam #ab4742, dil. 1:5000) overnight. Membranes were then washed three times with 1 × TBS/0.1% Tween-20, incubated with corresponding horseradish peroxidase (HRP) - conjugated antibody in 1% milk 1 × TBS/0.1% Tween-20 for 1 h. Then the membranes were again washed three times with 1 × TBS/0.1% Tween-20 and incubated with either Clarity ECL (Biorad) or Sirius ECL substrate (Advansta), and luminescence was assessed with LAS4000.

### Labile iron pool (LIP) measurement

A method based on calcein dequenching has been used [52]. Briefly, cells were incubated with 250 nM calcein acetoxymethylester-(calcein-AM) for 30 min in medium supplemented with 1% BSA but without serum and sodium bicarbonate. Samples were then washed twice with Hanks Balanced Salt Solution (HBSS). 10,000 cells were added to each well of a 96-well plate, and fluorescence measurement started at the excitation wavelength of 468 nm, emission wavelength of 517 nm, after initial 5 min measurement. 100 μM salicylaldehyde isonicotinoyl hydrazone (SIH) was added and the fluorescence was recorded after 2 min.

### ^55^Fe uptake measurement

Cells were dissociated by using the cell dissociating buffer (1% BSA, 1 mM EDTA, 1 mM EGTA), washed twice with the reaction buffer (50 mM HEPES (pH 7.4), 94 mM NaCl, 7.4 mM KCl, 0.74 mM MgCl_2_, 5 mM D-Glucose) and divided into Eppendorf tubes to contain 1 million of cells per sample with total volume of 200 μl. 1 μl containing 1 μCi of ^55^Fe in complex with citrate (1:10) was added. Cells were then incubated at 37°C for 90 minutes with occasional mixing and after the incubation, samples were cooled on ice. Background binding was determined by addition of 1 μCi of ^55^Fe to the cells followed by immediate cooling. Samples were then washed 5x with the reaction buffer, re-suspended in 100 μl of water and added to 5 ml of scintillation fluid. Samples were then measured on a scintillation counter and background corrected. Relative uptake was then calculated by comparing MCF7 spheres vs. control cells.

### ^55^Fe subcellular localization

Cells were incubated with 50 nM ^55^Fe complexed with citrate (1:10) for 72 hrs. Cells were then centrifuged and washed with 1x reaction buffer used in iron uptake experiments (50 mM HEPES (pH 7.4), 94 mM NaCl, 7.4 mM KCl, 0.74 mM MgCl_2_, 5 mM D-Glucose). Cells were counted and diluted with STE buffer (250 mM sucrose, 10 mM TRIS, 1 mM EDTA) to a concentration of 4 milion cells per 1ml STE. Cells were then disrupted as shown by Schmitt et al. to retain functional mitochondria [140]. Cellular homogenate was then spun at 800 × g for 5 minutes to collect nuclei then spun at 3000 × g for 5 minutes and resulting supernatant was spun at 9,000 × g for 10 minutes to gain mitochondrial fraction. Protein content in each faction was determined by the BCA-based assay and 20 μg of proteins was then measured on a scintillation counter and background corrected.

### Cellular viability assays (Cell Titer-Glo, Cell Titer-Fluor)

Cell viability assays were performed according to manufacturer's instructions (Promega, G7570 and G6080). Briefly, cells for the Cell Titer-Glow assay were seeded at of 5,000 cells per well into white luminescence plates, and after incubation with SIH, the Cell Titer-Glow reagent equal to the volume of medium was added and luminescence was captured using the TECAN 200 PRO reader. For the Cell Titer-Fluor assay, cells were seeded similarly but into a black fluorescent 96 well plates. After incubation with SIH, cells were incubated with fluorogenic peptideglycylphenylalanyl-aminofluorocoumarin and its fluorescence recorded at the excitation wavelength of 400 nm and emission wavelength of 505 nm using using Tecan infinity 200 PRO.

### Aconitase activity measurement

The Sigma aconitase activity assay (MAK051) was used. Absorbance was assessed at 450 nm according to manufacturer's instruction using Tecan infinity 200 PRO. The obtained values were corrected for the background activity of lysates without substrate and normalized to protein content assessed by the BCA method. Relative values compared to control cells were then plotted *via* Graphpad prism.

### Assessment of the IRE/IRP system activity via fluorescent EMSA

Cells were collected by centrifugation at 300 × g, 5 minutes, washed once with PBS a lysed in buffer containing 10 mM HEPES (pH 7.4), 3 mM MgCl_2_, 40 mM KCl, 1 mM DTT and 0.2% NP-40. Proteins were then quantified by the BCA method and 60 μg of protein lysate was then incubated with 4 μM of the Cy5 labeled IRE probe containing the 1× IRE sequence from the human FTH gene (Cy5-UCGUCGGGGUUUCCUGCUUCAACAGUGCUUGG-ACGGAACCGGCGCU) in 24 mM HEPES, 60 mM KCl, 5% Glycerol, 0.004 U/μl RNAsin, plus or minus 2% β-ME in a total volume of 20 μl for 20 minutes, then 2 μl of heparin [141, 142] was added and incubated for another 10 minutes. Consequently, 2.4 μl of 10× loading dye was added and the reaction mixture was loaded onto 3–20% AA gel in 1 × TBE. Electrophoresis was then run at 70 V for 30 minutes, followed by 120 V until the blue dye reached the bottom of the gel. The gel was then visualized by the Typhoon instrument.

### Mitochondrial respiratory complex I activity

Abcam mitochondrial respiratory complex I (CI) activity assay (ab109721) was used, utilizing immuno-capture of CI and then colorimetric reaction measuring its activity *via* absorbance increase at 450 nm according to manufacturer's instructions. Relative values compared to control cells were then plotted *via* Graphpad prism.

### Reduced glutathione (GSH) and reduced/oxidized glutathione (GSH/GSSG) ratio measurement

A fluorescence-based kit for determination of GSH and GSH/GSSG ratio was used according to manufacturer's instructions (BioVision). Briefly, cells were spun, washed with PBS and lysed in cell lysis buffer. 1 μg of total protein was loaded into 25 microliters of assay buffer in a 384-well plate. 25 microliters of the glutathione assay mixture (GAM) or total glutathione assay mixture (TGAM) mixture was added to samples, and reduced or oxidized glutathione standard curves plotted. Fluorescence was measured at the excitation wavelength of 480 nm and emission wavelength at 520 nm using Tecan infinity 200 PRO.

### Assessment of mitochondrial membrane potential (ΔΨmi) and reactive oxygen species (ROS)

To assess ROS or ΔΨmi, spheres and control cells were dissociated by cell dissociation buffer to obtain single cell suspension and incubated with fluorescent probes for 15 min. ΔΨmi was assessed with 50 nM tetramethylrhodamine methyl ester (TMRM), and ROS were evaluated using 5 μM dichlorofluorescein diacetate (DCF-DA), 2.5 μM dihydroethidium (DHE), 5 μM hydroxyphenyl fluorescein (HPF) or 2.5 μM MitoSOX. After incubation, cells were spun down and resuspended in PBS. Fluorescence was measured by flow cytometer (BD FACS Calibur) and expressed as a mean fluorescence intensity. Data were normalized to control cells and plotted *via* Graphpad Prism.

### Preparation of an acute promyelocytic leukemia (APL) *in vivo* model and isolation of LICs

An *in vivo* model of murine APL was prepared as previously described [139]. Briefly, leukemic spleen cells from hMRP8 PML-RAR transgenic mice were transplanted by retro-orbital injection into sub-lethally irradiated 12 week old FVB/N mice. Spleen cells from either leukemic or wild-type control mice were sorted using an Influx cell sorter based on expression of the following cell surface markers: *Sca*1 (clone D7; Biolegend), CD45/B220 (RA3-6B2; Biolegend), CD19 (MB19-1; Biolegend), CD3 (145-2C11, Biolegend) antigens, all pacific blue conjugated (omitting Gr1 antibodies from the usual depletion cocktail). The depletion cocktail-negative cells were then separated using antibodies against c-kit conjugated with allophycocyanin (clone 2B8; Biolegend) and CD34 conjugated with fluorescein isothiocyanate (RAM34; eBioscience). LICs from leukemic mice were characterized by depletion cocktail-, c-kit+, and CD34+ expression. Non-LICs from leukemic mice mice were characterized by depletion cocktail-, c-kit-, and CD34- expression. Counterpart non-leukemic populations were isolated from wild-type control mice.

## SUPPLEMENTARY MATERIALS FIGURES AND TABLES








