# Supplementary material for: Tumor-initiating cells of breast and prostate origin show alterations in the expression of genes related to iron metabolism
Source: Oncotarget. 2016 Dec 22;8(4):6376–98. doi: 10.18632/oncotarget.14093 (PMC5351639; doi:10.18632/oncotarget.14093)
Supplement: Supplementary file 1 [file oncotarget-08-6376-s001.pdf]

Tumor-initiating cells of breast and prostate origin show alterations in the expression of genes related to iron metabolism

Supplementary Materials

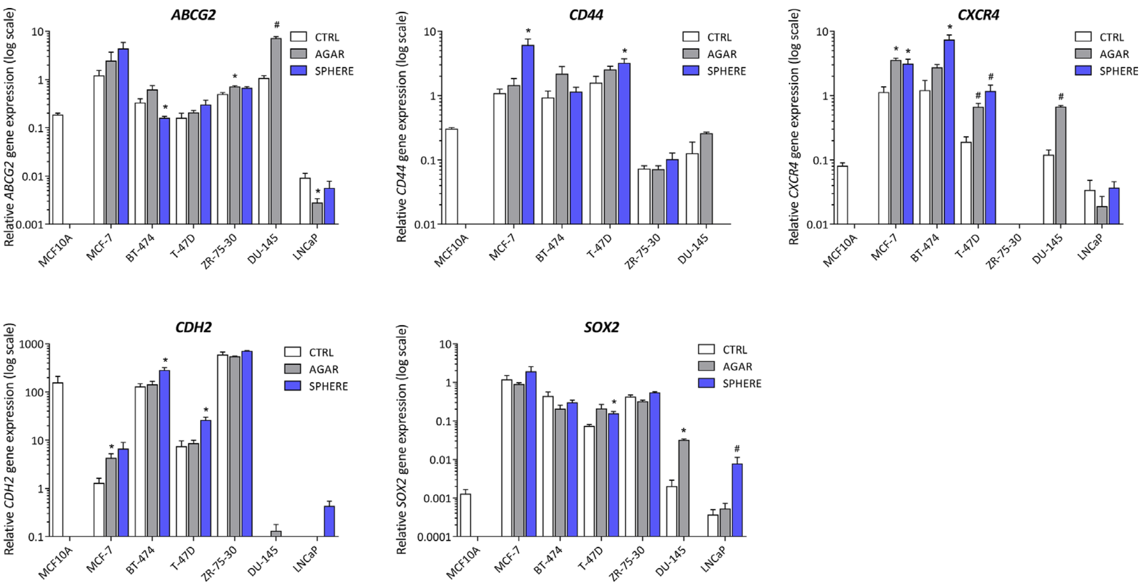

Supplementary Figure S1: Expression of stem cell and epithelio-mesenchymal transition (EMT) markers in various cell lines and their corresponding spheres representing the tumor-initiating cells (TICs). Expression profile of stem cell and EMT markers in non-malignant MCF10A, malignant breast MCF-7, BT-474, T-47D, ZR-75-30 and malignant prostate DU-145 as well as LNCaP cell lines. *P*-values were calculated by the GenEx software using unpaired *t*-test and were plotted using the GraphPad software. *P*-values lower than 0.05 are considered statistically significant and are denoted with a star symbol. The test was run using at least three independent biological samples and SEM are shown.

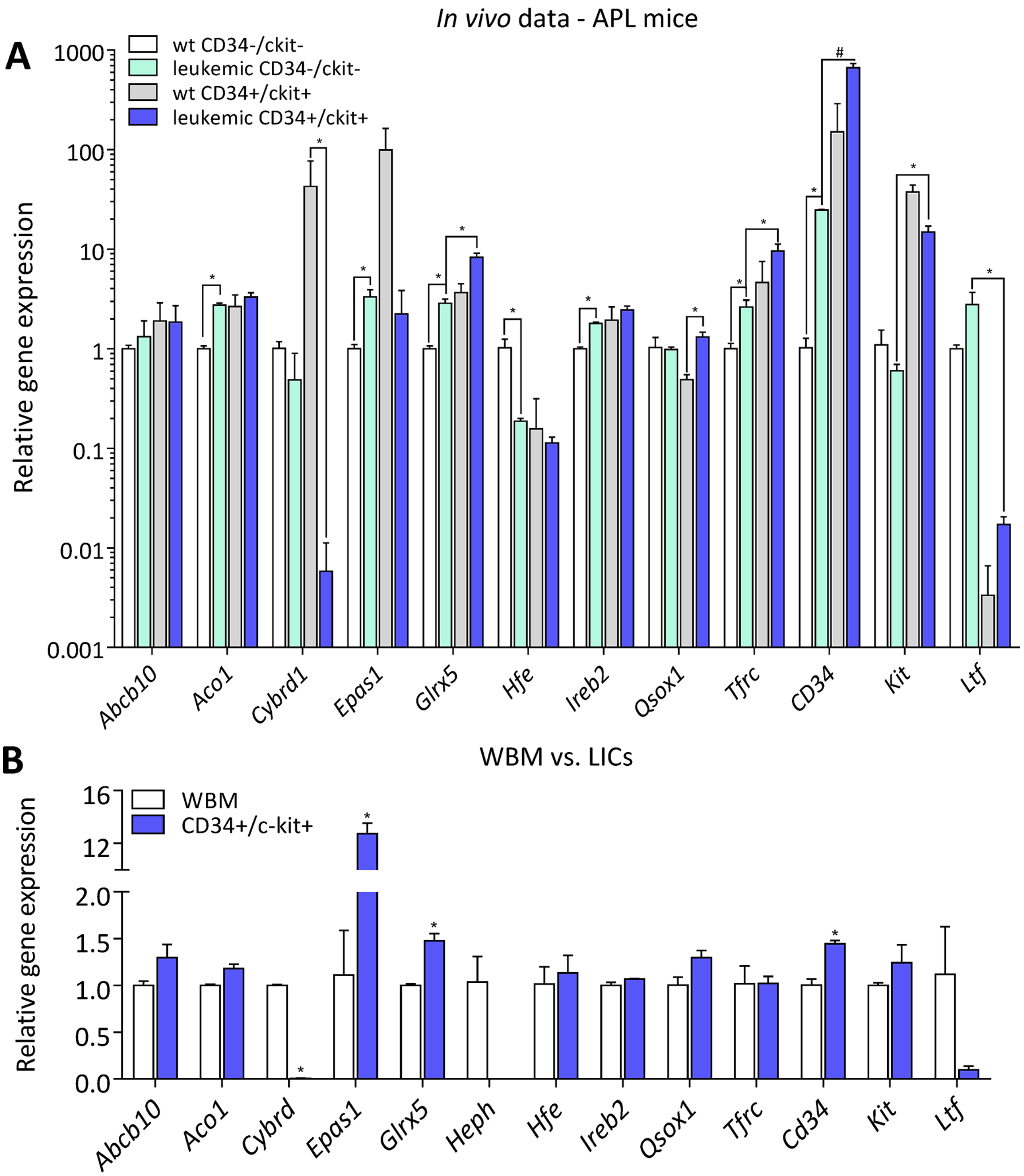

Supplementary Figure S2: Expression profile of selected iron metabolism-related genes at the *mRNA* level in leukemia-initiating cells (LICs) from an acute promyelocytic leukemia (APL) mouse model. Expression of selected iron metabolism-related genes (*Abcb10, Aco1, Cybrd1, Epas1, Glrx5, Heph, Hfe, Ireb2, Qsox1, Tfrc*) at the mRNA level in CD34+/c-kit+ (LICs) and CD34-/c-kit- (non-LICs) populations isolated from leukemic and healthy wild type (wt) mice (**A**). *Heph* expression is not plotted in panel A as there was no expression in any of the sorted populations. Comparison of the expression in the whole bone marrow (WBM) and sorted CD34+/c-kit+ (LICs) population in leukemic animals is also shown (**B**). Experiments were performed in biological duplicate, standard error represent SEM, *p*-values lower than 0.05 are denoted with a star and were calculated by the GenEx software using the unpaired *t*-test and plotted with GraphPad prism software. Number sign denote statistical significance with Dun-Bonferroni correction.

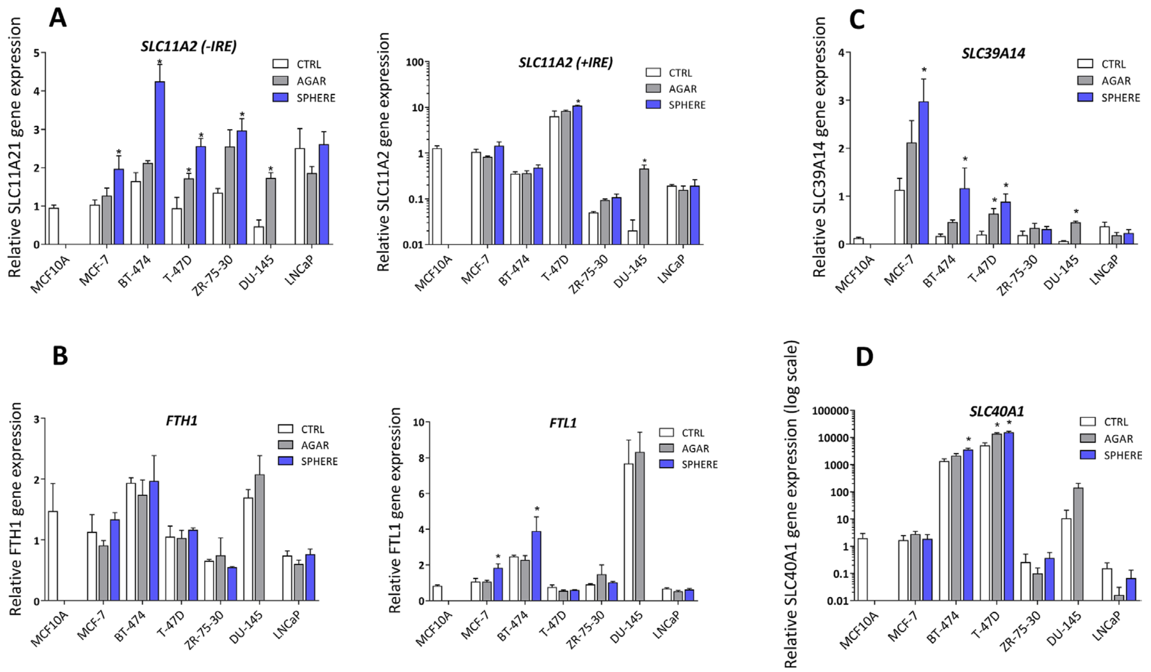

Supplementary Figure S3: Expression profile of additional genes regulating iron handling (solute carrier family 11 member 2; *SLC11A2*), storage (ferritin heavy and light chain; *FTH1*, *FTL1*), uptake (solute carrier family 39 member 14; *SLC39A14*) and export (solute carrier family 40 member 1; *SLC40A1*) at the *mRNA* level in tumor-initiating cells (TICs). The mRNA levels of additional regulators participating in iron handling (SLC11A2, panel **A**), iron storage (FTH1, FTL1, panel **B**), nontransferrin bound iron (NTBI) uptake (SLC39A14, panel **C**) and iron export (SLC40A1, panel **D**) were assessed by a qPCR analysis. Experiments were performed at least in triplicate, standard error is SEM, *p*-values lower than 0.05 are denoted with a star and were calculated by the GenEx software using the unpaired *t*-test and plotted with GraphPad prism software.

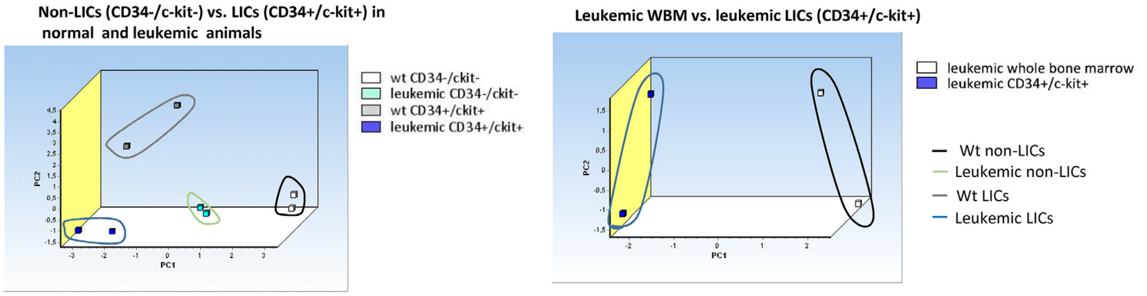

Supplementary Figure S4: Principal component analysis (PCA) based on iron metabolism-related genes (*Aco1*, *Abcb10*, *Cybrd1*, *Epas1*, *Glrx5*, *Hfe*, *Ireb2*, *Qsox1*, *Tfrc*) discriminates leukemia-initiating CD34+/c-kit+ cells (LICs) and CD34-/c-kit-(non-LICs) populations in the acute promyelocytic leukemia (APL) mouse model. Principal component analysis (PCA) based on selected iron metabolism-related genes was run on CD34+/c-kit+ and CD34-/c-kit- populations or whole bone marrow (WBM) populations using the GenEx software which was also used for plotting the PCA. White squares depict CD34-/c-kit- population from normal healthy animals, light grey show CD34+/c-kit+ population from normal healthy animals, light green denote CD34-/c-kit- population from leukemic animals, blue boxes show CD34+/c-kit+ population from leukemic animals. Second plot shows leukemic animals with white boxes depicting the whole bone marrow population and blue boxes show the CD34+/c-kit+ sorted population. Individual clusters were also highlighted with corresponding lines using identical colors.

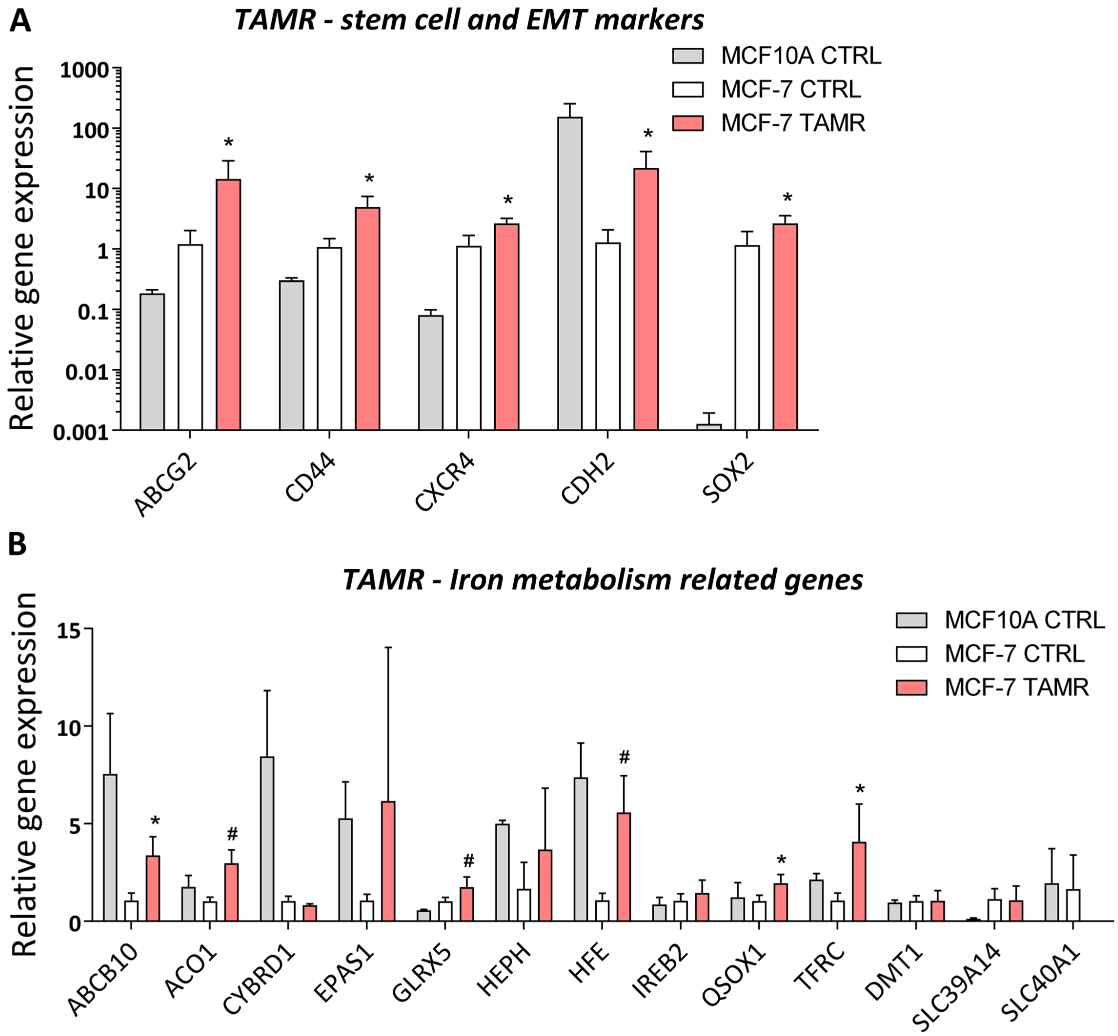

Supplementary Figure S5: Gene expression in tamoxifen resistant (TAMR) MCF7 cells. Expression of stem cell markers in the model of tamoxifen-resistant MCF7 cells is shown in (**A**). Iron metabolism-related gene with altered expression in mammospheres were also determined in the model of tamoxifen resistant cells, representing and alternative model of TICs (**B**). Experiments were performed at least in triplicate, standard error is SEM, *p*-values lower than 0.05 are denoted with a star and were calculated by the GenEx software using the unpaired *t*-test and plotted with GraphPad prism software. Number sign denotes statistical significance involving Dun-Bonferroni correction.

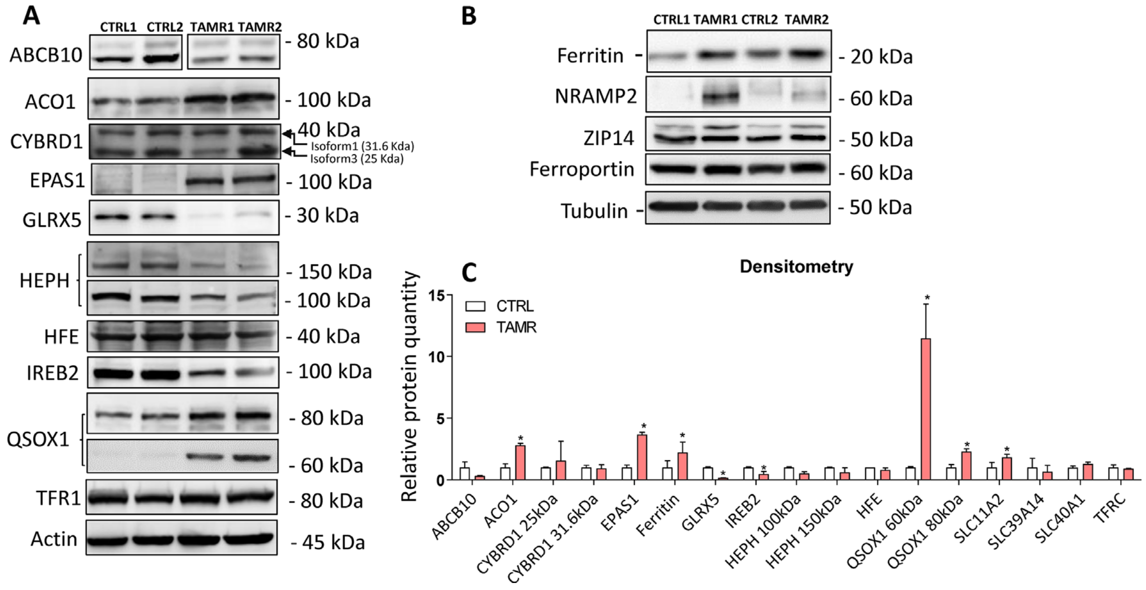

Supplementary Figure S6: Protein levels of genes with altered expression in tamoxifen resistant (TAMR) MCF7 cells. Altered genes identified by mRNA profiling in spheres samples of various cell lines were also determined on the level of corresponding protein in the model of tamoxifen resistant cells (**A**) together with additional iron-metabolism genes that are regulated on the protein level in the same model (**B**). Experiments were performed at least in triplicate, standard error is SEM, *p*-values lower than 0.05 are denoted with a star and were calculated by the GenEx software using the unpaired *t*-test and plotted with GraphPad prism software. Number sign denotes statistical significance involving Dun-Bonferroni correction. The protein expression in MCF7 cells grown as controls (CTRL), and tamoxifen resistant MCF7 (TAMR) shown in panel (**C**) was quantified by the image J software from 2 to 5 independent samples, standard error is SEM, *p*-values lower than 0.05 are denoted with a star and were calculated and plotted in GraphPad prism, using the unpaired *t*-test.

Supplementary Table S1: Expression profiling of iron metabolism-related genes in tumor-initiating cells (TICs) derived from various cancer cell lines. See Supplementary_Table_S1

Supplementary Table S2: Expression profiling of iron metabolism-related genes in leukemia-initiating cells (LICs) derived from the acute promyelocytic leukemia (APL) mouse model. See Supplementary_Table_S2

Supplementary Table S3: Raw data from FLUDIGM qPCR and additional qPCR data. See Supplementary_Table_S3
