# Supplementary material for: Tumor-initiating cells of breast and prostate origin show alterations in the expression of genes related to iron metabolism
Source: Oncotarget. 2016 Dec 22;8(4):6376–98. doi: 10.18632/oncotarget.14093 (PMC5351639; doi:10.18632/oncotarget.14093)
Supplement: Supplementary file 2 [file oncotarget-08-6376-s002.docx]

**Supplementary Table 1: Expression profiling of iron metabolism-related genes in tumor-initiating cells (TICs) derived from various cancer cell lines.**

|  | (MCF7 SPH) vs (MCF7 CTRL) | | (MCF7 TAMR) vs (MCF7 CTRL) | | (T-47D SPH) vs (T-47D CTRL) | | (BT-474 SPH) vs (BT-474 CTRL) | | (ZR-75-30 SPH) vs (ZR-75-30 CTRL) | | (DU-145 AGAR) vs (DU-145 CTRL) | | (LNCaP SPH) vs (LNCaP CTRL) | |
| --- | --- | --- | --- | --- | --- | --- | --- | --- | --- | --- | --- | --- | --- | --- |
| **Iron metabolism-related genes** | Fold change | *P-Value* | Fold change | *P-Value* | Fold change | *P-Value* | Fold change | *P-Value* | Fold change | *P-Value* | Fold change | *P-Value* | Fold change | *P-Value* |
| ***ABCB10*** | 1.928 | *0.017* | 3.260 | *0.003* | 1.724 | *0.150* | 1.713 | *0.155* | 1.860 | *0.029* | 2.399 | *0.045* | 1.341 | *0.085* |
| *ABCB6* | 1.862 | *0.044* | -1.220 | *0.137* | **1.344** | ***0.000*** | 1.447 | *0.054* | 1.076 | *0.803* | 1.817 | *0.009* | 1.189 | *0.119* |
| *ABCB7* | 1.363 | *0.017* | 1.420 | *0.107* | 1.853 | *0.025* | 1.518 | *0.013* | 1.389 | *0.143* | 2.052 | *0.078* | 1.159 | *0.363* |
| *ABCB8* | 1.395 | *0.130* | 1.007 | *0.966* | 1.456 | *0.076* | 1.314 | *0.026* | 1.040 | *0.861* | 1.935 | *0.021* | 1.039 | *0.742* |
| ***ACO1*** | **1.907** | ***0.001*** | **2.921** | ***0.000*** | 1.593 | *0.115* | 2.599 | *0.042* | 2.193 | *0.019* | 2.012 | *0.033* | 1.436 | *0.047* |
| *BMP6* | -1.108 | *0.792* | -3.291 | *0.023* |  | *NaN* | -1.510 | *0.470* |  | *NaN* | 4.104 | *0.045* | 1.520 | *0.142* |
| ***CYBRD1*** | 1.846 | *0.094* | -1.216 | *0.264* | 1.431 | *0.006* | 7.846 | *0.001* | 1.599 | *0.681* | 3.520 | *0.004* | 3.123 | *0.131* |
| ***EPAS1 (HIF2a)*** | 1.782 | *0.084* | 3.322 | *0.098* | 1.387 | *0.240* | 3.261 | *0.087* | 1.514 | *0.444* | 5.532 | *0.018* | 1.785 | *0.377* |
| *FTH1* | 1.309 | *0.319* | 1.363 | *0.366* | 1.175 | *0.385* | -1.026 | *0.908* | -1.188 | *0.031* | 1.204 | *0.368* | 1.020 | *0.896* |
| *FTL1* | 1.781 | *0.024* | 2.082 | *0.018* | -1.217 | *0.245* | 1.512 | *0.108* | 1.111 | *0.324* | 1.102 | *0.704* | -1.100 | *0.493* |
| *FXN* | -1.429 | *0.039* | 1.596 | *0.171* | -2.525 | *0.004* | -1.337 | *0.039* | 1.022 | *0.923* | -2.848 | *0.049* | -1.132 | *0.447* |
| *GLRX2* | -1.412 | *0.436* | -1.157 | *0.742* | 1.686 | *0.309* |  | *> 0.99* |  | *> 0.99* |  | *> 0.99* | -1.032 | *0.949* |
| ***GLRX5*** | **-1.845** | ***0.001*** | 1.691 | *0.023* | -3.670 | *0.023* | -2.523 | *0.083* | -1.242 | *0.538* | -1.143 | *0.639* | -1.492 | *0.242* |
| *HAMP* | 1.371 | *0.491* | 2.196 | *0.243* |  | *NaN* | 3.421 | *0.141* | 1.926 | *0.701* | -1.417 | *0.564* |  | *NaN* |
| ***HEPH*** | 3.653 | *0.123* | 2.783 | *0.290* | 3.660 | *0.003* | **6.837** | ***0.001*** | 1.116 | *0.913* |  | *> 0.99* | 1.938 | *0.061* |
| ***HFE*** | 1.653 | *0.058* | **5.367** | ***0.001*** | 1.592 | *0.017* |  | *NaN* | 1.008 | *0.960* | 2.123 | *0.020* |  | *NaN* |
| *HIF1* | -1.280 | *0.143* | 1.608 | *0.081* | 1.211 | *0.493* | -1.117 | *0.641* | 1.823 | *0.007* | 2.218 | *0.070* | 1.679 | *0.131* |
| *HMOX1* | 1.613 | *0.044* | 1.311 | *0.515* | 1.821 | *0.085* | 1.672 | *0.020* | 1.077 | *0.593* | 1.529 | *0.244* | 1.292 | *0.140* |
| *HMOX2* | 1.235 | *0.081* | -1.192 | *0.249* | -1.131 | *0.485* | 1.734 | *0.004* | 1.127 | *0.507* | -2.466 | *0.023* | 1.328 | *0.009* |
| ***IREB2 (IRP2)*** | 1.627 | *0.021* | 1.345 | *0.345* | 2.794 | *0.014* | 1.957 | *0.044* | 2.064 | *0.001* | 4.203 | *0.026* | 1.647 | *0.046* |
| *ISCA1* | -1.052 | *0.736* | 2.236 | *0.005* | 1.217 | *0.081* | -1.045 | *0.890* | -2.043 | *0.114* | -1.013 | *0.902* | -1.482 | *0.239* |
| *ISCA2* | -1.165 | *0.332* | 1.350 | *0.055* | -1.619 | *0.125* | -1.059 | *0.714* | -1.461 | *0.224* | -1.777 | *0.133* | -1.078 | *0.216* |
| *ISCU* | 1.162 | *0.382* | 1.162 | *0.387* | 1.180 | *0.027* | 1.278 | *0.082* | 1.095 | *0.455* | 1.168 | *0.326* | 1.184 | *0.191* |
| *LYRM4 (ISD11)* | -1.100 | *0.176* | -1.248 | *0.014* | -1.075 | *0.544* | 1.015 | *0.925* | -1.104 | *0.162* | -1.375 | *0.270* | 1.046 | *0.489* |
| ***QSOX1*** | **2.837** | ***0.001*** | 1.906 | *0.018* | **4.769** | ***0.000*** | **2.846** | ***0.000*** | 1.031 | *0.905* | 1.302 | *0.462* | 1.871 | *0.002* |
| *SLC11A2 (DMT1,NRAMP2 +IRE)* | 1.308 | *0.405* | 1.075 | *0.884* | 1.988 | *0.045* | 1.318 | *0.243* | 2.079 | *0.065* | 39.369 | *0.012* | -1.061 | *0.895* |
| *SLC25A28 (MFRN2)* | -1.016 | *0.933* | -1.042 | *0.886* | -1.031 | *0.864* | -1.103 | *0.624* | 1.124 | *0.061* | 1.325 | *0.416* | 1.103 | *0.607* |
| *SLC25A37 (MFRN1)* | -1.287 | *0.109* | 1.369 | *0.342* | -1.490 | *0.146* | -1.385 | *0.271* | -1.260 | *0.274* | -2.422 | *0.170* | -1.042 | *0.836* |
| *SLC48A1 (HRG-1)* | 1.329 | *0.291* | 1.211 | *0.669* | -1.217 | *0.541* | 1.550 | *0.126* | -1.874 | *0.594* | 1.998 | *0.170* | 2.351 | *0.163* |
| *STEAP3* | 1.258 | *0.448* | 2.056 | *0.042* | 1.214 | *0.613* | -2.513 | *0.045* | -1.190 | *0.732* | 1.607 | *0.291* | -1.534 | *0.428* |
| *TFR2* | 2.554 | *0.009* | -3.394 | *0.010* | 2.536 | *0.436* | -2.419 | *0.462* | -1.393 | *0.427* | 1.368 | *0.529* | -1.561 | *0.376* |
| ***TFRC (+IRE)*** | 2.113 | *0.004* | 3.716 | *0.007* | -1.080 | *0.811* | 1.955 | *0.086* | 2.460 | *0.036* | 4.037 | *0.055* | 3.066 | *0.006* |
| *TMPRSS6* | 1.705 | *0.288* | 3.367 | *0.035* |  | *NaN* | -1.853 | *0.336* |  | *> 0.99* |  | *> 0.99* | **31.664** | ***0.000*** |
| *VEGFA* | 1.430 | *0.478* | -1.227 | *0.743* | 3.278 | *0.004* | 1.282 | *0.325* | -1.355 | *0.486* | 1.989 | *0.004* | -1.068 | *0.868* |
| **Stem cell and EMT markers** |  |  |  |  |  |  |  |  |  |  |  |  |  |  |
| ***ABCG2*** | 2.768 | *0.142* | 10.110 | *0.007* | 1.847 | *0.159* | -2.008 | *0.041* | 1.333 | *0.119* | **6.844** | ***0.000*** | -1.749 | *0.269* |
| ***CD44*** | 5.093 | *0.002* | 4.574 | *0.003* | 2.171 | *0.049* | 1.270 | *0.530* | 1.337 | *0.439* | 2.616 | *0.120* |  | *NaN* |
| ***CXCR4*** | 2.743 | *0.026* | 2.591 | *0.032* | **6.052** | ***0.000*** | 6.962 | *0.012* |  | *NaN* | **5.765** | ***0.001*** | 1.744 | *0.442* |
| ***CDH2*** | 2.754 | *0.306* | 14.812 | *0.010* | 4.353 | *0.015* | 2.226 | *0.030* | 1.197 | *0.230* |  | *> 0.99* |  | *> 0.99* |
| *SOX2* | 1.472 | *0.385* | 2.526 | *0.039* | 2.118 | *0.003* | -1.361 | *0.479* | 1.282 | *0.187* | 19.104 | *0.002* | **18.685** | ***0.001*** |
