# Supplementary material for: Tumor-initiating cells of breast and prostate origin show alterations in the expression of genes related to iron metabolism
Source: Oncotarget. 2016 Dec 22;8(4):6376–98. doi: 10.18632/oncotarget.14093 (PMC5351639; doi:10.18632/oncotarget.14093)
Supplement: Supplementary file 3 [file oncotarget-08-6376-s003.docx]

**Supplementary Table 2: Expression profiling of iron metabolism-related genes in leukemia-initiating cells (LICs) derived from the acute promyelocytic leukemia (APL) mouse model.**

|  | (wt CD34+/ckit+) vs (wt CD34-/ckit-) | | (leukemic CD34-/ckit-) vs (wt CD34-/ckit-) | | (leukemic CD34+/ckit+) vs (wt CD34-/ckit-) | | (leukemic CD34+/ckit+) vs (wt CD34+/ckit+) | | (leukemic CD34+/ckit+) vs (leukemic CD34-/ckit-) | | (leukemic CD34+/ckit+) vs (leukemic WBM) | |
| --- | --- | --- | --- | --- | --- | --- | --- | --- | --- | --- | --- | --- |
|  | Fold change | *P-Value* | Fold change | *P-Value* | Fold change | *P-Value* | Fold change | *P-Value* | Fold change | P-Value | Fold change | P-Value |
| *Abcb10* | 1.62169 | *0.498567077* | 1.19127 | *0.750497086* | 1.64718 | *0.427922446* | 1.01572 | *0.985645531* | 1.38271 | *0.68387392* | 1.29235 | *0.156746489* |
| *Aco1* | 2.5403 | *0.098056561* | 2.76063 | *0.007423871* | 3.27161 | *0.011696898* | 1.28788 | *0.517117186* | 1.18509 | *0.279567551* | 1.17895 | *0.059866632* |
| *Cybrd1* | 24.67537 | *0.108561573* | -3.9381 | *0.396638518* | -480.2028 | *0.067739242* | -11849.18326 | *0.044002904* | -121.93766 | *0.150668485* | -257.33431 | 0.007345829 |
| *Epas1* | 76.37349 | *0.029941631* | 3.25464 | *0.031139566* | 1.58008 | *0.660025212* | -48.33512 | *0.080220603* | -2.05979 | *0.509769553* | 12.70655 | 0.032111939 |
| *Glrx5* | 3.56771 | *0.033239261* | 2.85304 | *0.013504638* | 8.21067 | *0.003558285* | 2.30138 | *0.079217968* | 2.87787 | *0.018205869* | 1.47427 | 0.019353173 |
| *Hfe* | -98.01971 | *0.313596945* | -5.34244 | *0.019297517* | -8.9383 | *0.01466507* | 10.96626 | *0.557383175* | -1.67307 | *0.086404645* | 1.11922 | 0.692584619 |
| *Ireb2* | 1.80563 | *0.266452907* | 1.79938 | *0.008156946* | 2.45377 | *0.012557405* | 1.35896 | *0.519183258* | 1.36368 | *0.085436299* | 1.06807 | 0.186875064 |
| *Qsox1* | -2.05979 | *0.132519794* | -1.01572 | *0.959429402* | 1.30586 | *0.455718244* | 2.6898 | *0.029174071* | 1.32639 | *0.168226599* | 1.29459 | 0.129755436 |
| *Tfrc* | 3.58631 | *0.231705802* | 2.59818 | *0.045831297* | 9.44794 | *0.008888094* | 2.63445 | *0.331126737* | 3.63637 | *0.033229528* | 1.01748 | 0.940421281 |
| *CD34* | 59.61072 | *0.124915418* | 24.67537 | *0.005621201* | 662.83226 | ***0.001593975*** | 11.11935 | *0.267594856* | 26.8621 | ***0.000890883*** | 1.44643 | 0.033762674 |
| *Kit* | 36.8221 | *0.016557665* | -1.68179 | *0.378058992* | 14.723 | *0.027683431* | -2.50099 | *0.058955698* | 24.76104 | *0.00460141* | 1.22901 | 0.318775476 |
| *Ltf* | -1533.38048 | *0.086945103* | 2.64818 | *0.102936629* | -59.19896 | ***0.00271965*** | 25.90215 | *0.296276913* | -156.76938 | *0.005624493* | -11.29412 | 0.065279469 |
