# Supplementary material for: Tumor-initiating cells of breast and prostate origin show alterations in the expression of genes related to iron metabolism
Source: Oncotarget. 2016 Dec 22;8(4):6376–98. doi: 10.18632/oncotarget.14093 (PMC5351639; doi:10.18632/oncotarget.14093)
Supplement: Supplementary file 4 [file oncotarget-08-6376-s004.docx]

**Supplementary Table 3: Raw data from FLUDIGM qPCR and additional qPCR data**

**MCF10A MCF-7 CTRL MCF-7 AGAR MCF-7 SPHERE MF7-7 TAMR BT-474 CTRL**

**BT-474 AGAR**

**BT-474 SPHERE T-47D CTRL T-47D AGAR T-47D SPHERE ZR-75-30 CTRL ZR-75-30 AGAR ZR-75-30 SPHERE DU-145 CTRL DU-145 AGAR LNCaP CTRL LNCaP AGAR**

**LNCaP SPHERE**

**Iron metabolism-related genes**

MCF10A 1 MCF10A 2 MCF10A 3 MCF7 C1 MCF7 C2 MCF7 C3 MCF7 C4 MCF7 C5 MCF7 A1 MCF7 A2 MCF7 A3 MCF7 A4 MCF7 A5 MCF7 S1 MCF7 S2 MCF7 S3 MCF7 S4 MCF7 S5 TAMR 3 TAMR 2 TAMR 1 BT474 C1 BT474 C2 BT474 C3 BT474 A1 BT474 A2 BT474 A3 BT474 S1 BT474 S2 BT474 S3 T47D C1 T47D C2 T47D C3 T47D C4 T47D C5 T47D A1 T47D A2 T47D A3 T47D A4 T47D A5 T47D S1 T47D S2 T47D S3 T47D S4 T47D S5 ZR7530 C1 ZR7530 C2 ZR7530 C3 ZR7530 A1 ZR7530 A2 ZR7530 A3 ZR7530 S1 ZR7530 S2 ZR7530 S3 DU145 C1 DU145 C2 DU145 C3 DU145 A1 DU145 A2 DU145 A3 LNcaP C1 LNCaP C2 LNcaP C3 LNCaP C4 LNCaP C5 LNCaP A1 LNcaP A2 LNCaP A3 LNcaP A4 LNcaP A5 LNcaP S1 LNcaP S2 LNCaP S3 LNCaP S4 LNCaP S5

***ABCB10*** 11.5485 9.5948 9.0521 11.7340 12.2126 15.0506 15.1955 11.2192 13.0861 12.5213 13.2092 13.5131 13.1583 12.9654 11.7102 13.3383 12.8334 10.7973 11.1297 13.2798 11.9311 14.5829 13.5290 14.1621 13.7348 13.7272 13.1897 12.5378 14.0611 12.4739 13.5071 15.8702 13.3959 14.6365 7.9020 12.9046 12.4058 13.1033 12.4790 13.1867 12.4469 11.9917 11.5461 12.7697 11.7261 13.3114 13.2991 22.3411 11.9136 11.2871 12.1315 11.6129 9.8983 12.1342 15.6841 12.9553 15.9028 11.8930 13.0352 12.9505 15.3549 12.7475 13.4905 12.6084 14.7477 13.4983 12.1792 13.7399 11.8552 13.1560 12.3297 12.4892 12.6244 13.2795 13.4390

*ABCB6* 10.1261 9.1436 8.8003 8.3428 8.3870 11.1618 11.5842 8.5803 9.5669 8.8440 9.4930 9.8041 9.6555 9.1519 8.9736 9.0880 10.2102 7.1154 9.9050 11.3105 10.6857 10.3068 10.1724 10.2323 10.7203 10.2920 9.6914 9.2469 9.6490 9.3442 11.0182 11.9939 10.9119 11.2898 6.8803 10.5783 10.2815 11.0685 10.5968 10.6028 10.0909 9.8129 9.5341 9.9570 9.6662 9.5617 10.4501 13.6504 9.0543 8.7649 9.5372 8.9390 7.3792 9.7983 11.3962 9.8249 11.8309 8.8649 9.5264 9.1988 11.8133 9.8723 9.9484 9.7238 10.5010 10.1912 8.9077 10.1589 9.0637 9.3170 9.4763 9.3358 9.5490 9.8349 9.7447

*ABCB7* 11.6535 9.7357 9.2368 10.6354 11.2230 13.0114 13.0690 10.3311 12.0199 10.9255 11.9630 12.1505 12.1994 11.9599 10.0088 12.3782 12.4316 10.2269 11.1105 12.9971 11.5457 11.8619 11.8632 11.6751 11.5613 11.8780 11.6387 10.7418 11.6509 10.3278 12.7623 15.0542 12.5889 13.9009 8.1162 11.8987 11.6115 12.3046 11.6513 12.0868 11.7160 11.2668 11.0901 11.7972 11.1995 11.6246 11.2349 20.7652 10.2092 9.6372 10.7946 10.2840 8.6618 10.3393 14.9192 12.1468 14.9133 11.2065 12.5712 12.2155 12.8378 11.0254 12.0116 11.2065 12.9624 12.6046 10.7540 12.4810 10.3165 11.3357 10.8658 10.7099 10.8142 11.8390 12.0824

*ABCB8* 13.7641 12.4433 12.0091 13.2526 13.6277 15.1453 16.0595 13.0822 14.2516 13.4584 14.4398 14.5691 14.4720 14.1186 13.5396 14.2269 15.0470 12.8039 14.0227 15.9553 14.8987 14.2826 14.3416 13.8390 14.0207 14.2944 14.6212 13.2277 14.1747 13.0055 16.0623 17.0031 15.5678 16.3686 10.6178 14.4701 14.4647 15.2121 14.6161 15.3913 14.5785 14.2499 14.4590 14.4825 14.2373 13.9159 13.7676 22.0117 13.4824 12.6215 13.8439 13.0390 11.1713 13.5607 16.3134 14.4306 16.5270 13.6703 13.9936 13.8735 15.9441 14.6480 14.8831 14.8236 15.4601 15.8408 14.4298 15.0181 13.9397 15.1236 14.2704 14.2512 14.4453 14.6068 15.2424

***ACO1*** 12.2079 10.4874 10.0275 10.6769 11.1943 13.4532 13.6803 10.2848 11.8363 11.0087 12.1903 12.2830 12.1463 11.7951 10.0265 12.2811 11.8350 9.6637 9.8655 11.9433 11.3339 14.4097 13.1073 13.9482 13.9606 13.5571 12.6454 11.6956 12.9844 11.7793 13.2879 14.8147 12.8490 14.1541 7.6853 12.1320 11.9605 12.4356 12.1890 12.4424 12.0868 11.6560 11.1919 12.2278 11.3664 11.8337 11.5552 20.4893 9.9627 9.2328 10.4455 10.0369 8.1567 9.9077 13.9964 11.6432 14.2131 10.8131 11.6859 11.4509 13.8281 11.8081 12.6232 11.8434 13.9320 13.3684 11.7022 13.3553 10.9970 12.0497 11.1980 11.0491 11.6789 12.3482 12.4818

*BMP6* 16.9580 21.8812 20.0676 17.1375 18.1883 20.7604 20.8928 17.0092 19.7763 19.0808 19.9971 19.7286 19.6093 18.7859 18.1387 19.1283 22.0066 17.6374 21.4164 20.8026 21.5372 22.4011 N/A 19.7852 21.2326 21.1467 20.6755 21.3459 N/A 21.4475 23.1579 N/A N/A N/A 20.0555 N/A N/A 25.6891 N/A N/A N/A N/A 24.8300 22.6148 N/A N/A N/A N/A N/A N/A N/A N/A 22.2979 N/A 23.9403 21.6244 21.9558 18.4097 20.0208 20.1024 19.0793 16.6981 16.4127 16.2234 17.6916 17.0236 15.7420 16.8132 15.4255 16.6344 16.5033 16.4412 15.7164 15.9497 15.8018

***CYBRD1*** 11.3103 9.4092 9.4362 11.9380 12.8189 14.6780 14.4875 12.3716 14.0828 12.4333 15.0759 14.5608 14.7552 13.4572 10.9343 14.2759 14.3962 9.7783 13.3327 14.9242 14.5737 15.8541 15.5120 16.3838 16.8828 15.8643 14.9610 12.1877 13.3474 12.4265 11.8540 12.7466 11.9479 12.3621 7.3794 11.3225 11.4547 11.8817 11.6485 11.4378 10.6582 10.6132 10.1724 11.1509 10.2100 24.6416 21.0453 N/A 22.6420 20.1368 24.2072 21.2384 20.5633 21.1145 15.4378 13.7639 15.8147 12.1829 12.2572 12.2532 N/A 23.2281 N/A 25.8431 N/A 22.9525 N/A N/A N/A 22.5060 20.0633 24.9751 22.0372 N/A N/A

***EPAS1 (HIF2a)*** 15.3176 13.5164 13.1259 15.4772 16.4818 17.7209 18.0641 14.8468 18.9368 17.3806 18.3946 18.7405 16.1112 16.5938 14.7916 16.2658 16.0997 15.6398 12.5030 16.8253 17.2375 20.3720 18.1588 19.2266 18.3782 18.2064 19.5805 16.6576 18.4433 16.6684 18.3010 19.0961 17.6920 19.1361 12.7947 16.6510 16.6194 18.4674 16.7515 18.0318 17.2484 16.2065 17.0799 16.8399 16.3858 20.6597 19.4623 N/A 18.6844 17.3316 19.6262 19.2889 17.1709 18.3445 19.6139 17.2031 20.5644 15.9168 15.5554 15.6294 20.3339 19.3453 21.1040 18.7222 23.7931 21.1939 20.0668 21.0934 18.7243 19.7723 18.2402 18.1829 18.6816 19.9493 21.3955

*FTH1* 4.7538 5.1245 4.8660 5.0877 4.9928 5.9471 6.6546 5.0669 6.3015 5.6876 6.3402 6.6680 6.0932 5.7543 3.9255 6.0343 6.5616 4.5018 5.1028 6.4227 5.9928 6.0580 6.3848 5.8864 6.7911 6.4250 6.0228 5.9887 5.8007 5.7768 8.2136 7.9875 7.2794 7.9251 4.1341 7.1346 7.1037 6.9961 7.2721 7.5823 6.8657 6.7725 6.4555 6.7784 6.6047 7.1577 7.2825 7.3251 7.1606 6.3637 5.7184 6.7836 5.2013 6.8368 7.0770 6.0549 7.4033 5.4944 5.7372 5.6243 8.3443 7.1703 7.6407 7.2960 8.1306 7.7929 7.2844 8.3963 6.9839 7.3002 7.2753 6.4220 7.1017 7.7749 7.1936

*FTL1* 8.7672 8.3678 7.6599 7.6242 7.3822 8.9011 9.4964 7.5728 8.3082 8.1132 8.7246 9.1936 8.8242 7.7277 6.3216 7.9998 9.0622 6.6706 7.0944 8.4007 8.1248 8.4619 8.5942 8.1393 8.6994 8.9471 8.3259 7.7718 7.4990 7.2632 10.8613 10.9765 11.1487 11.2135 6.8548 10.9483 10.8183 10.7213 10.6702 10.5674 10.4299 10.3999 9.9343 10.6936 10.1097 9.3181 9.4298 8.1726 8.8036 7.8826 7.4290 8.6158 6.9884 8.4776 7.3767 6.8691 7.8041 6.0436 6.4821 6.2292 11.2168 9.9099 10.3124 9.9593 11.0275 11.0989 10.1416 11.1308 9.6808 9.8505 10.3586 9.5511 10.0365 10.4364 10.0632

*FXN* 18.0953 17.0896 15.9882 17.0491 16.4658 19.7176 19.8484 16.6275 18.9125 18.2615 19.2519 19.1345 18.5400 18.5902 17.6562 19.4138 20.0722 17.5185 18.0659 18.6767 17.2683 19.0804 19.2141 18.7204 19.9663 17.9961 19.6834 19.0881 19.3967 18.9152 19.5870 19.7161 20.4981 19.7658 15.6637 20.3016 20.0926 20.1531 20.1119 20.7653 19.9455 20.2756 20.3203 20.3680 20.1017 17.9913 18.8411 N/A 17.9136 17.8903 18.1063 17.3321 16.3322 17.9055 17.6346 17.8739 18.0059 18.0009 18.6447 18.5218 19.7480 18.5744 18.7859 18.5718 19.4839 18.5346 17.6384 18.8191 17.9151 18.0775 18.4208 18.8205 17.9745 18.9380 19.2354

*GLRX2* 26.3686 23.3108 24.7386 23.5739 21.7894 24.9147 26.3739 21.4792 23.9783 25.3939 24.0607 24.9562 24.0244 25.0407 23.3429 25.0504 24.0853 24.1040 23.6814 25.5664 24.5046 N/A N/A N/A 25.5437 24.6690 29.0822 N/A 26.8755 26.9587 25.8770 28.8713 26.0022 24.3846 21.4277 25.1600 27.0535 24.9612 25.6820 25.2659 24.7041 24.0045 23.6058 N/A 25.1953 N/A 26.6406 N/A 25.6961 23.1675 23.9601 24.5369 23.3428 24.5433 N/A 26.8486 N/A 25.3677 N/A 25.6082 27.6960 26.3519 N/A 27.0007 25.0701 27.9457 26.2622 26.7057 24.1140 24.5555 25.2233 26.5788 N/A 24.9924 27.3850

***GLRX5*** 23.7668 22.8280 22.4526 21.4017 21.0480 23.7329 24.1019 21.6066 23.3049 23.1910 23.4464 23.2948 23.2320 23.6200 22.5165 24.3537 24.3631 22.4233 21.6041 23.4017 22.0632 23.6037 24.2261 22.9464 25.4994 23.2955 23.4484 24.0536 24.5362 25.3197 23.7765 24.7118 23.4747 25.3347 18.7146 24.2455 23.5495 23.7264 24.1394 25.9839 24.4786 24.6507 23.8097 24.5142 27.0342 24.3318 24.2474 N/A 24.6947 23.2528 23.6996 23.2691 22.3373 24.6181 24.9989 24.6281 25.2150 23.7810 24.2216 24.5411 26.9583 23.9070 25.5987 24.0790 26.5087 25.1474 23.7385 25.2441 24.6508 24.5921 24.8658 25.4398 24.2071 26.2660 26.4870

*HAMP* N/A N/A 27.8010 24.2373 23.3265 N/A N/A 24.2939 24.7121 24.5084 24.4656 24.9504 24.3445 25.6397 24.3754 26.4421 24.6835 23.8847 25.1822 24.5497 24.8220 N/A 26.6327 28.1704 N/A 25.1206 28.9158 24.1936 25.3979 26.5936 N/A N/A N/A N/A N/A N/A N/A 28.5887 N/A N/A N/A N/A N/A N/A N/A 27.6450 29.2926 N/A 24.7763 24.3424 30.3286 24.8393 23.8759 30.3296 N/A 24.5896 26.5405 23.5515 26.2486 26.2072 N/A 26.3224 N/A N/A N/A 26.5396 N/A N/A N/A N/A 26.5766 24.1889 N/A N/A N/A

***HEPH*** 17.1622 21.1702 20.5567 N/A 22.1275 N/A 23.6378 25.3896 23.0144 22.6792 23.6258 25.0991 N/A 24.7246 19.9667 N/A N/A 21.4367 20.7860 24.9608 23.1868 23.6002 N/A 24.3068 N/A 21.5873 21.8729 20.8026 21.5673 20.2977 23.6511 22.8572 22.2691 23.1836 17.4501 23.1425 21.0074 22.3172 22.9005 22.0130 20.5485 20.2042 19.5277 19.9889 18.8806 22.3952 19.9085 N/A 20.7316 17.1624 20.8272 21.3859 18.7447 19.2660 N/A 24.3547 N/A 22.1462 N/A 24.4355 21.7912 18.8603 19.9697 18.9707 20.5525 20.7453 19.6559 21.7022 18.7244 18.9117 17.9525 16.8720 18.9293 19.8322 19.1135

***HFE*** 14.1042 13.2283 12.2109 14.8280 15.5256 18.6947 17.4272 14.7443 16.5259 15.5970 16.0933 16.2902 16.8249 16.2677 14.2419 16.4571 16.5237 15.0728 13.2231 15.6463 14.7985 N/A N/A 24.3436 N/A 21.6668 23.4339 N/A N/A 23.7349 12.7926 13.1205 12.8815 13.0018 7.7291 11.5155 12.1413 11.4858 11.5104 11.9498 11.1929 11.0554 10.9039 11.2706 10.8472 13.8720 13.6837 21.8555 12.8427 12.0797 12.9763 13.0720 11.3041 13.3401 15.8444 15.1710 15.9144 13.1124 13.9265 13.7567 N/A N/A N/A N/A N/A N/A 33.3553 N/A N/A N/A N/A 30.0165 30.7508 N/A N/A

*HIF1* 16.1637 15.6200 15.1738 15.7040 16.1364 18.6181 18.1348 15.4329 17.7293 16.2205 17.6357 17.4721 17.8698 17.1650 15.9402 18.4556 18.6208 16.5920 15.3282 18.1195 17.1212 16.8595 17.7077 16.7696 17.1512 16.9739 17.9062 16.5973 17.9461 16.4002 19.3003 21.7094 18.7885 20.3125 14.5738 19.1392 18.2301 19.8605 18.3095 19.8977 18.7054 18.2878 18.6411 18.7762 17.9936 18.2124 18.4807 22.6290 16.8991 16.8108 16.4339 16.7973 15.3529 16.7067 19.7038 16.9563 18.5595 15.7080 16.7020 16.4845 21.9568 18.9626 19.8622 19.5716 20.0958 19.5396 18.3866 19.4971 17.7335 19.7216 18.2885 18.1774 17.6983 19.5220 20.3534

*HMOX1* 17.4196 18.8366 18.5037 17.0273 16.6162 18.3456 18.5686 16.4190 18.2033 16.9181 18.5646 18.6244 17.3601 17.4281 15.1225 17.9598 18.3407 15.8705 15.7329 19.3799 18.2513 19.7193 19.7846 18.9953 18.8787 18.8309 19.2583 18.1926 19.2359 17.9855 19.5384 19.9409 18.8813 20.4857 13.4189 18.2020 18.2868 18.7242 17.8989 18.4518 17.7802 17.4608 16.9146 17.3757 17.5367 17.4275 18.0034 15.6775 16.8501 15.7457 14.9408 16.9176 15.1551 17.1701 16.7129 16.4366 17.0436 15.0875 15.1840 15.2170 19.9161 17.5769 18.7087 17.8443 19.3773 18.7671 17.7435 18.2740 17.4129 17.7431 17.3589 16.8949 17.8636 18.6930 18.1059

*HMOX2* 12.9722 12.3388 11.7542 10.8507 10.9197 13.3868 14.1991 10.8427 12.6483 12.1580 13.2685 13.2055 12.7895 12.1922 10.6992 13.1243 13.1590 10.4721 12.0042 13.8669 13.2158 14.0537 13.7279 13.6839 14.1295 13.8680 13.1803 12.4944 13.2387 12.4767 13.9137 15.0478 14.0488 14.4611 8.9840 13.6868 13.6038 14.1155 13.8029 14.1852 13.7109 13.3502 12.6216 13.8470 12.9112 13.1421 13.9432 23.9667 12.8248 12.1743 13.1184 12.7073 10.9678 12.8520 14.7095 14.2581 14.8724 14.1063 15.3995 15.3650 15.6224 13.6847 14.2787 13.8202 15.3496 14.3648 13.2447 14.6114 13.3841 14.2315 12.9709 13.2577 13.7469 13.9849 14.0790

***IREB2*** 11.9170 10.4474 9.3859 9.1547 9.9858 12.5003 12.0634 8.6804 10.7459 9.9484 10.8158 10.8891 10.9792 10.5166 8.8076 11.1052 10.7413 8.6716 9.8921 12.1670 10.2961 11.9208 11.8199 11.7989 10.9016 11.4807 11.0111 10.1848 11.7414 9.8340 12.9807 15.1749 12.5850 14.0553 7.4247 11.6410 11.1462 11.8601 10.9037 11.3429 11.1728 10.6173 10.4271 11.4577 10.2326 13.2222 13.2231 24.0320 11.3954 10.8642 11.7935 11.4438 9.8968 11.6074 15.3341 12.1548 15.6373 10.6148 11.7479 11.6725 13.4157 11.4027 12.3248 11.1324 13.4030 12.5228 10.7596 12.4793 10.2322 11.3074 10.2221 10.2966 10.9283 11.8043 12.1564

*ISCA1* 15.5545 15.8550 14.9935 13.4059 13.2786 15.3167 15.2450 13.1698 15.8267 13.8646 15.1066 15.2556 14.8051 14.8244 12.6888 15.4709 15.6449 13.2692 12.6310 14.7526 13.6950 15.3326 16.8921 15.9024 16.1855 15.5832 17.1222 15.6012 16.4965 15.3580 15.7363 16.4190 15.5540 15.9309 11.3975 15.5300 15.1567 15.8810 15.6745 15.8636 14.5193 14.7942 14.6291 14.4019 14.3879 16.9449 15.9395 26.9478 17.2128 15.6455 16.8341 16.7181 15.2414 16.8857 15.4987 14.1795 15.7638 13.3360 14.6688 14.6448 15.6901 13.7600 14.2105 14.5321 15.0443 15.8149 15.0806 15.7089 14.6125 14.9453 14.2169 13.4321 13.8128 15.7353 16.2093

*ISCA2* 15.1043 15.0510 14.2260 13.2693 12.9814 15.9530 15.9219 13.3024 14.0604 13.9918 14.6365 15.2729 15.2666 14.7337 13.7212 15.1153 16.1583 13.7835 13.9995 15.3312 14.4431 14.9984 15.7601 15.1769 16.2738 14.5645 15.8727 14.8595 15.5105 14.9413 15.7224 14.6567 14.7416 14.7870 10.4857 14.7465 14.6993 14.7796 14.8082 15.5060 14.6204 14.5866 14.7808 14.1225 14.8544 15.5499 15.6577 15.9524 15.3850 14.8691 13.8482 15.4027 13.4145 16.0522 15.7529 15.5326 15.8289 15.0872 15.9707 15.6714 17.5303 15.6654 16.0065 15.6478 16.6412 16.5253 15.1417 16.2368 15.1842 15.6599 15.7119 15.4764 15.6549 16.1799 16.3380

*ISCU* 10.3311 9.1840 8.6655 8.8668 8.9394 10.7729 11.0965 8.8861 10.4087 9.6316 10.5045 10.5425 10.0340 10.4435 8.1569 10.3249 10.6067 8.9159 9.1910 10.7259 10.7774 11.4410 11.2242 11.0651 11.6323 11.4422 10.9734 10.6400 10.9385 10.2183 10.7497 11.7985 10.6480 11.3711 6.4233 10.4733 10.1507 11.4502 10.2239 11.4742 10.0261 9.6921 9.5808 9.8926 9.7013 11.9385 11.7930 21.0349 11.2964 10.8659 11.7593 11.0579 9.4632 11.1007 12.5218 11.3219 12.4946 10.2087 11.5115 11.0710 12.6572 10.6738 11.0834 10.9328 12.2543 11.7128 10.1998 12.1008 10.3423 11.1179 10.5929 10.0088 10.4266 11.3015 11.3856

*LYRM4 (ISD11)* 8.9916 8.0215 7.4509 8.3514 8.2507 10.8675 10.9494 8.1149 9.8809 9.2674 10.1595 10.2059 9.9504 10.1396 8.6684 10.3723 10.6776 8.3346 9.5123 10.8493 10.7265 9.4195 9.9316 9.3159 9.6849 9.7868 9.5460 9.3430 9.4598 8.9292 9.7494 10.1324 9.8395 9.8696 5.7445 9.1968 9.1636 9.7262 9.2524 9.6570 9.1520 8.9402 8.7410 9.2330 8.8856 9.6084 10.0057 18.5188 9.1865 8.4732 9.6418 9.1204 7.7427 9.4033 9.8031 9.3524 10.1087 8.3359 9.8579 9.5735 11.3655 9.3399 9.8262 9.4577 10.6475 10.2975 9.0404 10.3563 9.0128 9.6332 9.4270 9.1498 9.2715 9.8107 9.9811

***QSOX1*** 9.4913 10.1859 9.7050 9.1457 9.7866 11.0161 11.7971 8.8122 10.2986 9.2307 9.9649 10.7473 9.7759 8.8935 8.2928 9.1525 9.8750 7.7915 9.1296 10.8892 9.7332 11.9320 11.9008 11.6402 11.7111 11.5258 12.1071 9.7857 10.4459 9.8428 13.2395 15.5735 12.5608 14.2263 8.7221 11.6547 11.6286 12.1485 12.0008 10.7318 10.4953 10.2083 10.3760 11.0778 9.9943 11.2611 10.6376 21.4667 10.5191 9.1427 10.7420 10.2179 8.4621 10.4509 12.5239 10.1980 12.6611 10.5987 10.4408 10.3243 11.1171 9.7520 10.2342 9.8267 10.7381 10.8804 9.5962 10.3707 8.9735 10.1566 8.2161 8.1821 8.8553 9.4011 9.8231

*SLC11A2 (NRAMP2, +IRE)* 12.0340 11.9671 11.2220 11.1419 12.2855 13.4133 13.3353 11.1799 13.0653 12.0914 13.4407 13.3937 13.2827 13.4624 10.3845 13.7265 12.7849 10.0315 11.5966 15.1306 11.9824 15.0393 15.5824 15.2520 15.4883 15.0626 15.6219 14.2653 15.6164 13.9241 11.5089 14.0544 11.7587 13.0279 6.5395 10.7981 10.8847 11.0722 10.9048 10.8262 10.4576 10.3764 9.9602 10.2895 9.9472 17.6160 17.6779 23.7727 16.0341 15.3280 16.4363 15.7655 13.8849 16.5397 23.1075 17.6612 21.6993 14.6556 14.5946 14.4457 17.7852 15.4804 16.0185 15.6507 16.8623 18.6771 15.8801 16.7949 14.6688 15.8533 14.9621 14.3032 14.9674 17.0205 18.2992

*SLC25A28 (MFRN2)* 14.8464 14.4999 13.9350 14.6453 14.2028 16.8617 17.3021 14.7669 16.2054 15.2799 17.0144 16.4015 15.4339 16.5643 14.7497 17.0742 16.0753 14.3979 14.9680 16.6084 17.4768 16.5440 15.7515 15.8864 16.8805 16.4692 15.5417 15.6238 16.4820 15.6263 15.8255 16.6489 15.9406 15.9785 10.9721 15.9114 15.5029 15.9673 15.4766 16.3229 15.3545 15.0327 14.0068 15.4430 14.8432 17.3016 17.6203 21.3369 16.8278 15.5353 16.3711 16.4918 15.0049 16.7960 16.9331 16.6946 17.2234 14.5530 16.0178 16.1871 19.5836 17.0114 17.6380 17.4475 18.6956 17.2845 16.6918 18.0304 16.9851 17.7041 17.0265 16.4046 17.9528 18.1126 17.5004

*SLC25A37 (MFRN1)* 16.8721 17.1075 16.6040 17.8086 17.7243 20.3715 20.1474 17.8743 21.1512 18.8176 20.2662 20.3557 19.6586 20.0114 17.4754 20.2602 20.7169 18.2531 19.3673 19.4096 18.4303 19.6782 20.9240 19.8171 19.4695 19.6025 21.2712 19.8428 20.8938 20.2196 20.2196 20.2771 19.3847 19.9604 15.3194 21.1587 19.9618 21.0238 19.8291 20.4519 18.6017 20.4189 18.9550 19.4426 19.7175 19.4584 19.2261 21.5539 19.1454 18.6697 18.9967 18.6472 17.5386 19.2590 17.7351 18.1347 17.5506 16.9653 19.0007 18.4074 20.7033 18.1553 18.9509 18.5293 20.7925 18.8340 18.2850 19.3317 17.6942 18.4208 18.8737 18.2743 18.7541 19.5142 19.3415

*SLC48A1 (HRG-1)* 23.8131 19.7368 20.4681 19.1647 19.2026 21.4525 20.3696 19.4258 20.7975 19.5172 21.8222 21.2374 22.0626 20.7012 18.4340 20.2191 20.9550 18.2225 18.5852 20.8524 21.7097 21.2599 20.9935 19.9664 21.0741 19.6606 20.2621 19.4400 20.4868 19.5239 22.2264 21.1855 20.1521 21.6560 15.8982 20.1418 19.8937 20.4378 20.2437 22.2463 20.3676 20.6771 20.2921 20.0451 20.2510 21.3590 23.8825 17.4659 22.2645 22.9003 17.2270 24.7228 20.4529 21.8211 20.8234 21.2624 22.6258 18.8140 19.7458 20.2801 22.6426 22.1830 21.1852 24.0343 25.3763 22.1493 21.4940 23.1984 22.3136 22.9924 20.3369 20.4976 22.2104 21.7365 21.8042

*STEAP3* 21.6991 19.5700 19.7642 21.2506 21.3961 22.8009 22.6763 20.3149 22.9240 22.5191 22.4643 23.9541 23.9043 22.1487 19.6764 22.0808 23.8149 20.1383 20.0884 22.4946 21.6477 24.6022 24.9226 23.3578 23.2284 22.6152 24.4493 N/A N/A 25.3354 24.2865 25.6939 23.0124 25.0800 17.8786 25.2890 22.6826 22.3216 23.5116 N/A 23.2347 22.7683 22.1384 23.0394 22.4661 23.0968 21.8339 N/A 23.4779 21.7658 22.3591 22.5282 20.1232 21.9176 25.1441 23.2220 24.7093 23.1654 22.3565 22.6835 25.6863 21.8807 23.4890 22.8418 22.5482 23.6494 22.6759 22.3705 22.2650 22.6496 22.1171 24.9448 22.9119 24.4435 22.4677

*TFR2* N/A 30.1076 N/A 20.6728 20.9301 24.2563 23.6639 21.2712 23.5619 21.5067 22.3026 22.5066 23.1831 21.3210 21.1348 21.6794 22.1428 19.1006 27.3867 N/A 24.3933 22.4470 30.9426 25.6564 23.4829 26.8374 23.8178 25.0436 26.6568 23.7483 29.0437 30.8721 25.3686 22.4866 20.4821 26.8091 25.2272 25.9261 26.0967 25.2672 23.5402 23.2246 26.7052 23.7533 23.9791 26.7092 26.1536 N/A 27.7242 25.8598 N/A 26.3357 25.1496 N/A 21.8620 21.9697 24.2723 20.9612 21.4396 21.5373 23.7496 21.9897 23.3250 21.7767 23.9898 22.8109 21.0538 22.4167 20.7460 21.2306 22.6141 21.2511 22.2845 23.4531 25.7625

*TFRC (-IRE)* 13.9395 13.2009 12.4681 13.2635 13.9472 15.7806 16.8240 12.7464 14.5197 14.3016 14.3246 15.3779 14.7036 13.9940 12.3105 14.9232 14.8217 13.7660 12.4486 14.9135 13.1271 15.4118 14.4391 14.7911 14.0662 13.7489 13.4370 13.2925 15.0752 12.8919 13.9544 15.3689 13.1309 14.6813 8.9981 12.3780 11.8557 13.9887 12.5673 11.1377 13.4528 12.7686 13.1106 13.4586 12.8546 15.5542 15.9889 N/A 13.7385 13.6187 13.4227 13.8420 12.6888 13.9659 17.8980 14.0474 17.3862 12.5297 14.0717 14.1685 17.1525 14.5411 14.5470 13.9204 16.3309 14.9007 13.4677 14.3168 12.3235 13.8943 12.5810 12.9189 12.4396 13.9410 15.2040

***TFRC (IRE)*** 14.1237 13.2025 12.4552 13.3003 13.9680 15.8003 16.9504 12.7526 14.7003 14.2024 14.4925 15.3358 14.6226 14.1651 12.1071 14.8031 14.6707 12.5960 12.3504 14.9296 12.9097 15.4545 14.6153 14.7588 14.1846 13.9029 13.3893 13.3340 14.8396 12.8812 13.8743 15.3986 13.1659 14.7207 7.9727 12.3512 11.8505 12.6782 12.5532 11.2713 13.4922 12.5450 12.7377 13.2960 12.7124 15.4157 16.1763 N/A 13.7957 13.6416 13.5463 13.7291 12.6517 13.5275 17.9941 14.2435 17.7266 12.6042 14.1800 14.2635 17.4041 14.6313 14.7627 14.0731 16.2996 15.0342 13.4163 14.5093 12.3204 13.8875 12.4450 12.8499 12.4992 13.8412 14.7838

*TMPRSS6* 21.8530 22.4289 20.4157 18.6671 18.2575 21.1382 21.3011 20.7226 21.7051 20.4704 19.2524 19.8914 18.6319 18.0806 19.1764 20.1020 20.8739 18.9791 18.2243 19.3190 19.4656 22.8349 20.9194 22.1671 22.5438 30.5546 22.3966 21.7269 22.7009 23.2909 N/A 22.6052 N/A N/A 21.5436 N/A 22.4646 24.8507 N/A 23.0940 N/A N/A 22.5809 N/A N/A N/A N/A N/A N/A N/A N/A N/A 22.3771 25.7611 N/A N/A N/A 23.2567 21.3747 22.2574 24.0938 20.1641 22.3723 22.3509 24.8668 23.4956 23.3098 23.6838 20.8063 19.9573 15.0853 15.6386 18.4487 18.7997 18.2819

*VEGFA* 10.6870 11.5921 10.3153 10.6035 10.8676 10.6040 12.0706 10.4353 12.1330 11.0174 11.8659 12.4747 12.0350 11.3490 7.5210 12.0683 12.0885 9.9422 9.6988 13.5616 12.5811 11.1643 12.3016 11.3071 10.1456 12.0848 11.8541 10.9410 11.4758 10.4082 13.6460 15.0874 13.1275 14.4798 7.8848 12.5714 12.2311 12.8265 12.0330 9.6813 10.5135 11.1295 11.1988 10.9934 10.9235 12.3133 11.0706 22.0773 10.9821 10.1008 11.7585 11.3166 9.9167 11.5741 12.3627 10.4993 12.3358 9.1899 10.2390 9.9168 13.6188 11.3940 13.4008 10.3461 13.6415 14.3086 12.6380 13.5953 10.3524 10.0983 11.8659 11.1670 11.0669 12.9210 13.1823

**Stem cell and EMT markers**

***ABCG2*** 17.2031 16.6009 15.6501 13.1692 12.8861 16.4480 16.9162 11.7522 14.8088 13.8893 14.5040 14.9976 12.1234 14.2026 14.4428 13.2371 12.9242 10.0043 9.2071 12.6232 13.0767 17.8598 16.9777 17.4403 17.3155 16.5465 16.0800 17.7843 18.9591 17.6839 19.5624 21.2298 18.7102 19.7929 13.8976 17.8853 17.5883 18.5194 18.3579 18.8961 18.9093 16.7665 16.5363 17.4745 18.1666 16.0568 16.5729 23.7866 15.0177 14.4770 15.4955 15.3886 13.4642 15.2644 16.8439 14.8248 17.0431 11.6514 12.9924 12.8755 N/A 24.0755 23.0707 21.8198 N/A 23.8105 25.2443 23.6671 22.7774 N/A 23.2883 23.3849 23.1346 24.5777 21.8549

***CD44*** 13.2320 12.7924 12.2014 9.5510 10.5528 13.6114 12.3766 9.9574 11.6357 10.5547 11.8010 12.3227 10.4600 8.4137 9.2657 9.2188 10.0506 8.3258 9.2630 10.8356 9.1583 13.6115 12.3672 12.8677 11.1354 12.8932 11.6558 11.7838 13.4541 11.7020 12.4543 14.7956 12.9615 13.7782 7.5430 11.3969 11.7042 12.0698 10.9611 11.8999 12.2662 10.5917 10.3087 11.3950 10.4763 15.7511 16.3683 N/A 15.7763 14.3886 15.7418 15.3425 13.6117 14.3840 17.7853 14.2020 17.8731 13.8472 14.4641 14.5109 N/A N/A N/A N/A N/A N/A N/A N/A N/A 21.1964 N/A N/A N/A N/A N/A

***CXCR4*** 14.1600 13.4430 13.5356 8.2910 8.9691 12.9083 12.1798 8.8960 8.7022 7.8269 9.2122 9.5363 9.5138 8.9428 9.3325 9.2923 9.6813 7.6862 8.4803 10.2418 10.1126 12.5405 10.7514 11.9528 10.8016 10.5207 10.1016 8.5995 8.6353 8.7391 15.3775 15.6728 13.7414 15.5466 11.2807 12.7011 12.5758 12.9820 12.8425 11.7865 11.7497 11.5450 12.0669 10.6498 11.7206 N/A 21.2381 N/A N/A 22.0994 N/A N/A N/A 24.7390 16.1028 13.8416 16.3344 11.2529 12.4314 12.1423 17.4948 16.3316 19.6288 14.8031 20.4330 18.5369 17.9355 20.2683 16.3701 15.6909 16.3406 16.5119 15.5115 17.8320 15.8161

***CDH2*** 11.4163 12.0326 11.7516 16.9901 18.1110 22.5907 20.6514 16.7990 17.5133 18.1861 17.0197 18.6238 17.1112 16.1669 18.8044 16.8845 16.8829 20.0647 13.5426 16.1908 17.8952 13.4714 14.3017 13.0215 13.8519 13.8203 13.0271 12.1655 12.2662 12.0272 17.4134 21.7154 17.4517 18.6342 15.3302 18.6174 16.8434 18.5570 17.4215 17.1546 15.3473 15.8595 15.3895 17.1706 15.2666 10.9786 10.7871 19.9865 10.3059 9.6447 10.6432 9.8312 8.3052 10.1499 N/A 21.1417 N/A 24.0704 22.6492 N/A N/A N/A 22.4808 N/A N/A 23.3215 N/A N/A N/A N/A 20.3104 20.5888 23.0682 22.9359 N/A

*SOX2* 23.6930 21.3910 20.6362 11.6563 12.0737 12.4627 13.9685 11.4908 12.6929 11.9694 13.0585 13.7814 13.7044 12.2579 9.1725 12.7411 13.5142 12.1461 10.3659 12.6620 12.1630 15.0018 16.3126 14.2927 15.7126 17.1565 16.3029 15.7046 15.5221 14.8413 18.3443 19.4492 18.1327 19.0627 13.5425 16.8836 16.1833 16.3820 15.2623 18.3025 16.7365 16.5059 16.7704 16.0455 16.1681 14.3272 14.9920 N/A 14.3631 13.5309 14.8575 13.6940 11.8355 13.7910 22.7485 22.9902 24.4793 17.8955 18.5124 18.8808 25.0237 26.4492 N/A N/A N/A 25.5480 N/A 24.3240 22.4916 24.9551 18.6742 22.3805 21.4867 21.7651 21.2354

**Normalization genes**

*GAPDH* 5.4996 5.0737 4.6829 4.8809 5.0474 7.2932 7.1741 4.8997 5.8878 5.4857 6.2650 6.5866 6.7216 6.3235 5.2412 6.6990 7.3614 4.6657 5.9884 7.4625 7.1118 6.7505 6.9136 6.4116 7.0058 7.2054 6.8008 6.5391 6.7641 6.1370 8.3345 9.2618 8.2739 8.7461 4.3442 7.8638 7.8432 7.8670 7.9244 7.1543 7.9928 7.7490 7.5316 8.0386 7.8321 5.6351 5.7189 13.7054 5.0939 4.6888 5.7679 5.3512 3.9736 5.6508 7.5506 6.0043 7.8565 6.0802 6.5668 6.3770 7.3156 6.2064 6.6091 5.9618 6.5421 7.2399 5.8821 6.5664 5.3742 5.5847 6.0554 5.9398 5.8442 6.0978 6.1917

*HPRT1* 9.2311 8.2035 7.6195 6.7770 6.7596 9.9319 9.6275 6.6924 8.0230 7.4940 8.3970 8.5815 8.5504 8.4763 7.5482 9.1743 9.4828 7.1043 8.4113 9.5749 8.9449 10.6622 10.6978 10.1225 10.7405 10.8511 10.6569 10.3855 10.8444 10.1611 10.6821 11.6633 10.7128 11.2220 6.6455 10.3535 10.0744 10.8022 10.4169 10.6488 10.5379 10.7211 10.2379 10.6337 10.3320 8.4926 8.9697 14.3270 7.7214 7.4250 8.1918 7.8919 6.3794 8.2150 10.1649 8.9384 10.5731 8.4656 9.9614 9.8556 12.0226 9.6935 9.9576 9.5176 11.2766 10.2929 8.7265 10.4616 8.6381 9.2874 9.4319 9.4625 9.5020 10.0527 9.9826

*POLR2A* 8.7876 7.9808 7.4002 7.4793 7.7774 10.3616 10.7068 7.0394 9.4212 7.8919 8.9820 8.9460 9.3154 8.8751 7.3216 9.5159 9.6646 7.0706 7.6383 9.4480 8.7555 9.4913 9.5214 9.5024 9.2645 9.3361 9.8201 8.3919 9.8154 8.2125 10.1624 12.0934 9.8700 10.9700 5.6611 9.4019 8.8125 9.6588 9.3303 9.2360 8.8128 8.6352 8.4981 8.8404 8.3016 9.5038 9.6300 18.2794 9.4445 7.9340 8.5532 8.2415 6.7079 7.9413 12.1813 9.5623 12.3467 9.0628 9.1814 9.1819 11.5032 9.7087 10.1460 9.5807 10.7917 10.7290 9.4772 10.2835 9.0215 10.2879 9.0511 9.3880 9.8602 9.7742 10.2441

*PPIA* 13.9468 14.4806 13.8360 13.4117 13.3291 16.1388 15.9057 13.0557 14.7004 13.9603 15.2704 14.9749 15.3607 15.5169 13.8634 15.3247 15.5003 13.2877 13.9527 15.7037 16.0517 14.4544 14.2434 14.1978 14.6047 14.5728 13.9697 13.6150 13.8865 13.3855 14.7284 15.5080 14.3346 15.0030 10.6454 13.9403 13.8906 15.3530 14.1885 14.3070 13.8132 13.8020 13.6809 14.0797 13.8482 14.1054 13.9273 22.4826 13.0730 12.7188 13.8966 13.4538 11.5582 13.6426 15.5267 14.0305 15.4970 13.3857 14.6486 14.4930 14.7753 12.8472 13.4006 12.9643 14.0330 13.9339 12.3782 13.9284 12.0946 12.8349 12.7619 12.4969 13.0453 13.3322 13.6076

*RPLP0* 5.6482 5.2658 4.8606 4.8830 4.7204 6.5792 6.9673 4.9117 5.9438 6.0341 6.7364 6.7967 6.4897 6.1055 4.6928 6.8663 6.9273 5.1911 5.4102 6.4521 6.5956 6.8964 6.9828 6.6100 7.0868 6.9060 6.6625 6.7230 7.2228 6.7471 6.7820 6.7527 6.7543 6.8717 4.3092 6.6288 6.6581 7.2846 6.8324 6.8662 6.6491 6.9553 6.4087 6.8677 6.4978 7.0878 6.9963 15.1017 6.6327 5.9570 7.0731 6.9290 5.3436 7.0933 6.9579 6.8152 7.2142 6.2932 6.9413 7.1396 6.9887 5.9325 6.3325 6.0178 6.5899 6.8883 6.1014 6.4888 5.9450 6.3461 6.1628 6.0068 6.4284 6.6322 6.5426

*TBP* 13.3536 12.4141 11.6708 11.1375 11.3343 13.4791 13.9277 10.9609 13.3702 11.9958 13.3602 13.2510 13.3135 13.2167 10.4734 13.8768 13.5017 10.8137 12.3904 14.2828 13.3828 14.2037 14.4451 14.2260 14.3507 14.3185 14.2609 13.3921 14.3239 13.4152 14.6766 16.0753 14.4526 15.2569 9.2744 14.3708 13.7434 14.7735 14.0755 14.3668 13.6319 13.5021 13.0953 13.8103 13.2255 14.1710 14.7689 20.9567 13.4343 12.9724 13.5142 13.3058 11.7928 13.7266 15.4907 14.0433 15.7771 12.8301 14.6061 14.5509 16.5082 14.0230 14.6461 14.0305 16.0523 15.0798 13.6400 15.5874 13.6714 14.3963 13.8144 13.6547 14.3548 15.1743 14.9541

**Genes excluded because of high Cq, high variability or bad efficacy of the assay**

*HFE2*

*SLC40 (FPN1) HIF3A*

*FTMT HAMP*

**Genes assessed separately and without pre-amplification**

*SLC39A14* 34.3120 32.6005 31.9805 29.0979 30.0809 31.3841 29.8147 29.1367 29.3378 28.3245 28.8313 28.9610 29.7869 28.5256 28.7482 28.6552 29.6032 28.1506 29.0092 29.9813 N/A 33.5046 31.8699 33.4373 31.1274 31.6052 31.3247 30.1004 32.2696 29.2942 32.7875 31.3847 35.0392 33.9065 31.3433 30.3215 31.4044 30.9704 30.4378 30.7408 31.5792 29.9558 30.5549 30.9158 30.0367 35.0001 33.6165 40.1012 32.1864 32.6732 32.0559 32.8573 32.2592 32.1139 33.2809 35.6706 35.2352 31.1990 31.9869 31.4967 34.2477 32.7339 32.7722 32.4861 33.7808 31.8650 31.4685 32.6998 32.6256 33.6086 31.8755 30.7994 30.9256 32.3609 36.4677

*SLC40A1* 34.8410 35.4881 35.6665 35.0000 35.7387 37.9922 37.1951 33.6376 35.0499 33.7514 34.6710 35.1922 34.9835 37.7547 35.9432 36.0439 35.9748 34.0387 N/A N/A N/A 25.9046 25.0865 26.6579 25.4120 25.2608 24.9565 24.3665 25.6514 24.2557 23.4066 23.2931 26.1873 24.8376 22.8283 22.6033 22.4937 22.2848 22.0854 22.0457 22.4076 22.0852 22.5510 22.7844 22.3953 44.5449 N/A N/A 38.4638 42.7007 40.9111 N/A 38.1381 37.2443 30.7819 38.6578 38.7201 29.0499 31.4278 28.5161 N/A 38.3405 N/A N/A 38.3600 N/A 39.8101 N/A N/A N/A N/A N/A 37.1233 N/A N/A

Table shows raw Cq values of a panel of selected genes, separated into iron metabolism-related, stem cell and epithelia-mesenchymal transition (EMT) marker related and reference genes. A list of genes that were included but were excluded due to high Cq or bad efficacy is listed together with additional qPCR data. It is important to note that the FLUIDIGM analysis included 18 cycles of pre-amplification and thus Cq levels are lower while additional qPCR data show Cqs without pre-amplification.
